# Effects of eHealth interventions on psychological outcomes of post intensive care syndrome-family: a systematic review and meta-analysis

**DOI:** 10.3389/fmed.2026.1847415

**Published:** 2026-06-10

**Authors:** Baolin Huang, Jiaqi Li, Kaibo Shen, Sheng He, Ruoyu Luo, Yangyang Wang, Na Yin, Jiyong Jing, Ju Zhang

**Affiliations:** 1 School of Public Health and Nursing, Hangzhou Normal University, Hangzhou, Zhejiang, China; 2 School of Nursing, Zhejiang Chinese Medical University, Hangzhou, Zhejiang, China; 3 Zhejiang Provincial People's Hospital, People's Hospital of Hangzhou Medical College, Hangzhou, Zhejiang, China

**Keywords:** eHealth interventions, meta-analysis, Post Intensive Care Syndrome-Family, psychological well-being, systematic review

## Abstract

**Introduction:**

eHealth interventions represent a novel approach to improving Post Intensive Care Syndrome-Family. However, the effectiveness of these interventions on psychological outcomes in the families of ICU patients remains uncertain.

**Objective:**

The purpose of this study was to examine the effectiveness of eHealth interventions in alleviating psychological symptoms (anxiety, depression, and post-traumatic stress disorder) of Post Intensive Care Syndrome-Family.

**Methods:**

We systematically searched the databases across Cochrane Library, PubMed, Web of Science, Embase, and CINAHL from 1st January 2012 to 27th June 2025. Randomized controlled trials of eHealth interventions aimed at improving Post Intensive Care Syndrome-Family were included in the review.

**Results:**

This systematic review included 11 randomized controlled trials, involving 1,200 participants. The pooled results demonstrated that eHealth interventions did not significantly alleviate anxiety, depression, or PTSD symptoms in family members of ICU patients within 3 months of follow-up. However, subgroup analyses indicated that eHealth with early-stage psychological support led to a statistically significant improvement in depression symptoms (SMD = −0.32, 95% CI: −0.62 to −0.01).

**Conclusion:**

eHealth interventions did not demonstrate statistically significant short-term (≤3-month follow-up) improvements in psychological symptoms of Post Intensive Care Syndrome-Family. Integrating early-stage psychological support into eHealth interventions may have a potential benefit in improving the psychological symptoms of ICU patients’ families, although further rigorous validation is needed.

**Systematic review registration:**

https://www.crd.york.ac.uk/PROSPERO/view/CRD420250656227, Identifier: CRD420250656227.

## 1 Introduction

Having a family member hospitalized in an Intensive Care Unit (ICU) is a highly stressful event that can significantly impact the psychological well-being of relatives, increasing their risk of developing anxiety, depression, and post-traumatic stress disorder (PTSD) (1, 2). This cluster of adverse psychological symptoms in relatives has been defined as Post Intensive Care Syndrome-Family (PICS-F), which possibly impacts their life for years (3–5). Previous studies reveal that the prevalence of anxiety, depression, and PTSD among ICU patients’ relatives was approximately 40, 23, and 35%, respectively (4). Furthermore, these symptoms often co-occur. A 12-month multicenter cohort study found that 38.3% of ICU patients’ family members experienced at least one psychological symptom (anxiety, depression, or PTSD), and 22.8% experienced two or more symptoms (6). Family members are not merely ICU “visitors” (7); they often serve as primary supporters and caregivers for critically ill patients during hospitalization and throughout recovery, forming a closely interconnected patient–family dyad (8, 9). Consequently, poor psychological health among family caregivers not only diminishes their own quality of life but may also impair their caregiving capacity and is associated with worse patient outcomes, including increased readmission risk (10–12). Therefore, the psychological well-being of ICU patients’ family members deserves greater attention, and evidence-based interventions aimed at improving their psychological outcomes represent an urgent priority.

Previous reviews (12–14) have shown that family-centered interventions, such as family meetings and open visitation policies in the ICU, strengthen family engagement and support, which are considered beneficial for alleviating the psychological disorders of family members of ICU patients. These interventions usually require face-to-face implementation. However, during the COVID-19 pandemic, hospitals worldwide were forced to implement strict visitation policies for ICUs to mitigate the spread of the virus and protect vulnerable patients (15). These restrictions on interpersonal interactions made it difficult to implement family-centered interventions and negatively affected families’ psychological well-being (16). In response, remotely accessible eHealth interventions have emerged as an innovative alternative to reduce care interruptions for ICU patients’ families, maintain communication, and alleviate their psychological burden (15, 17). eHealth, defined as the use of information and communication technologies (ICT) for health (18), supports: (i) the storage, management, and transmission of health data; (ii) clinical decision-making; and (iii) the facilitation of remote care (19, 20). It encompasses a wide range of technologies, including video conferencing, mobile applications, virtual reality (VR), and online platforms (17). The integration of eHealth into mental health services is increasingly prevalent, representing a growing trend (17, 21).

As an emerging approach to address PICS-F, eHealth interventions have been developed to target key needs of ICU patients’ family members, including timely information and communication with clinicians, as well as emotional and psychological support, all of which are closely linked to psychological well-being (16, 22). However, evidence regarding the effectiveness of eHealth interventions in alleviating psychological symptoms among family members of critically ill patients remains inconsistent. While some studies (23, 24) have reported improvements in anxiety, depression, and PTSD symptoms, others [e.g., Cox et al. (25); Drop et al. (26)] have not observed significant benefits. These discrepancies may be partly attributable to heterogeneity in intervention strategy (information and communication-focused vs. psychologically informed support) and in the timing of initiation (during ICU hospitalization vs. post-discharge). Such heterogeneity, coupled with the increasing adoption of eHealth approaches in critical care settings, underscores the need for a systematic review.

Despite a consensus on the importance of improving ICU families’ psychological outcomes (16, 27, 28), the specific interventions recommended for this purpose remain unknown (1, 12). Therefore, this systematic review focuses on eHealth interventions, aiming to evaluate their effectiveness in alleviating psychological symptoms of ICU patients’ families, explore the characteristics of effective eHealth interventions through comparison, and provide further reference for developing intervention strategies to strengthen the psychological well-being of ICU patients’ families in the post-pandemic era.

## 2 Methods

This study was reported following the Preferred Reporting Items for Systematic Reviews and Meta-Analyses (PRISMA) (29) and was registered in the International Prospective Register of Systematic Reviews database with the registration number (CRD420250656227).

### 2.1 Information sources and search strategy

Given the formal proposal of the term PICS-F by the Society of Critical Care Medicine (SCCM) in 2012 (5), a systematic search was carried out in Cochrane Library, PubMed, Web of Science, Embase, and CINAHL from 1st January 2012 to 27th June 2025. The complete search strategy can be found in the Supplementary Table S2.

### 2.2 Inclusion and exclusion criteria

The inclusion and exclusion criteria were developed and applied according to the PICO framework (Participants, Interventions, Comparators, and Outcomes). Detailed eligibility criteria are outlined in the Supplementary Table S3.

#### 2.2.1 Participants

This review included studies focusing on adult family members or informal caregivers (aged ≥18 years) of adult patients admitted to the ICU for at least 24 h. Studies targeting the families of pediatric, neonatal, or prenatal ICU patients were excluded.

#### 2.2.2 Interventions

Eligible studies used eHealth interventions as the primary treatment for the experimental group. The interventions were required to: (i) deliver core content via electronic media (e.g., telephone, computer, smartphone, or tablet), and (ii) allow remote access, with no restrictions to healthcare facilities.

Studies were excluded if: (i) the intervention was delivered face-to-face or through printed materials without the use of electronic media; (ii) electronic media was used but restricted to healthcare settings without remote access; or (iii) the intervention consisted solely of pre-recorded, non-interactive video/audio materials.

#### 2.2.3 Comparators

Comparator conditions included: (i) no eHealth intervention (e.g., face-to-face interventions or print material-based interventions); (ii) pre-recorded, non-interactive video/audio materials; and (iii) usual care, placebo, waitlist, or no intervention. Studies were excluded if the comparator group also met the inclusion criteria for eHealth interventions.

#### 2.2.4 Outcomes

Studies were required to report at least one psychological outcome (anxiety, depression, or PTSD) of ICU patients’ families, using standardized and validated scales. Studies not reporting any psychological outcomes of ICU patients’ families were excluded.

#### 2.2.5 Study designs

Only randomized controlled trials (RCTs) with full-text articles published in peer-reviewed journals were included. Systematic reviews, meta-analyses, conference abstracts, study protocols, and incomplete articles were excluded. No language restrictions were applied.

### 2.3 Study selection and data extraction

After deduplication, two reviewers (Baolin Huang and Jiaqi Li) independently screened the titles and abstracts of potentially eligible studies. Only articles meeting the predefined inclusion criteria were retained for full-text review and subsequent data analysis. Any discrepancies or disputes encountered during the screening process were resolved through discussion with a senior reviewer (Ju Zhang) to achieve consensus.

Data extraction was conducted independently by two reviewers using a standardized form, with discrepancies adjudicated through consensus involving a third reviewer. The extracted details consisted of: (i) study characteristics (first author/publication year; country; sample size; age). (ii) eHealth intervention characteristics (content, dose, delivery format). (iii) outcome data (measurements and scores for anxiety, depression, and PTSD).

### 2.4 Risk of bias and certainty of the evidence

The Revised Cochrane Risk-of-bias Tool for Randomized Trials (RoB 2) (30) was used by two reviewers (Baolin Huang and Jiaqi Li) independently to assess methodological quality and risk of bias of the included studies. We considered the risk of bias from the following domains: the randomization process, deviations from intended interventions, missing outcome data, measurement of outcomes, selection of the reported result, and overall bias. Each domain was algorithmically categorized as “low risk,” “some concerns,” or “high risk” based on signaling questions, with an overall risk judgment synthesized from all domains. Disputes were resolved through discussion to reach a consensus.

The certainty of evidence was evaluated using the Grading of Recommendations, Assessment, Development, and Evaluation (GRADE) approach (31), which evaluates factors including study design, bias risk, inconsistency, indirectness, and imprecision. Evidence quality was classified as high, moderate, low, or very low. The evidence derived from RCTs was initially rated as high-quality but downgraded based on these factors.

### 2.5 Data synthesis and analysis

Given the substantial variability in follow-up time points across studies (ranging from 3 days to 12 months post-intervention) and that most studies (7/11) reported outcomes at 3 months, we extracted outcome data at the 3-month follow-up or, when unavailable, at the closest available time point within 3 months. This approach aimed to provide a relatively consistent estimate of the short-term effects of eHealth interventions while retaining the available data for quantitative synthesis. However, due to the limited number of included studies, we did not further stratify the analyses according to different follow-up time points within the first 3 months, which may still have introduced temporal heterogeneity into the pooled estimates.

Meta-analyses were conducted using the “metan” package in Stata 18.0. Intervention effects were calculated as standardized mean differences (SMDs) with 95% confidence intervals (CIs), accounting for differing outcome measurement scales across studies. Heterogeneity was assessed using the Chi-squared test (the *p*-value) and *I*^2^ statistic. The *I*^2^ statistic ranges from 0–40%, indicating negligible heterogeneity; 30–60%, representing moderate heterogeneity; 50–90%, indicating substantial heterogeneity; and 75–100%, reflecting considerable heterogeneity. A random-effects model was applied when *I*^2^ exceeded 50%, while a fixed-effects model was used for *I*^2^ values below 50% (32). Sensitivity analyses were performed using both the leave-one-out approach and by excluding studies with high attrition rates (≥20%) (33). Publication bias was evaluated with Egger’s test when at least ten studies were included (34). Statistical significance was predefined at a *p*-value < 0.05.

Subgroup analysis was conducted according to the characteristics of the intervention, including interaction modes, support strategies, and initiation timing. (i) Interaction modes: eHealth interventions were categorized as synchronous interaction (real-time communication), asynchronous interaction (delayed communication) (35), and hybrid interaction. (ii) Support strategies: the support strategies of eHealth interventions were classified as psychological support or information and communication support. Specifically, interventions involving psychological treatments or psychotherapy, such as cognitive behavioral therapy or mindfulness therapy, are coded as psychological support, while those providing structured medical information or facilitating communication with healthcare professionals are coded as information and communication support. (iii) Initiation timing: the differences in the initiation timing of intervention within psychological support interventions or information and communication support interventions were examined. Interventions initiated during ICU hospitalization were categorized as early-stage interventions, while those initiated after ICU discharge were categorized as late-stage interventions.

## 3 Results

### 3.1 Literature screening process

**Figure 1:**
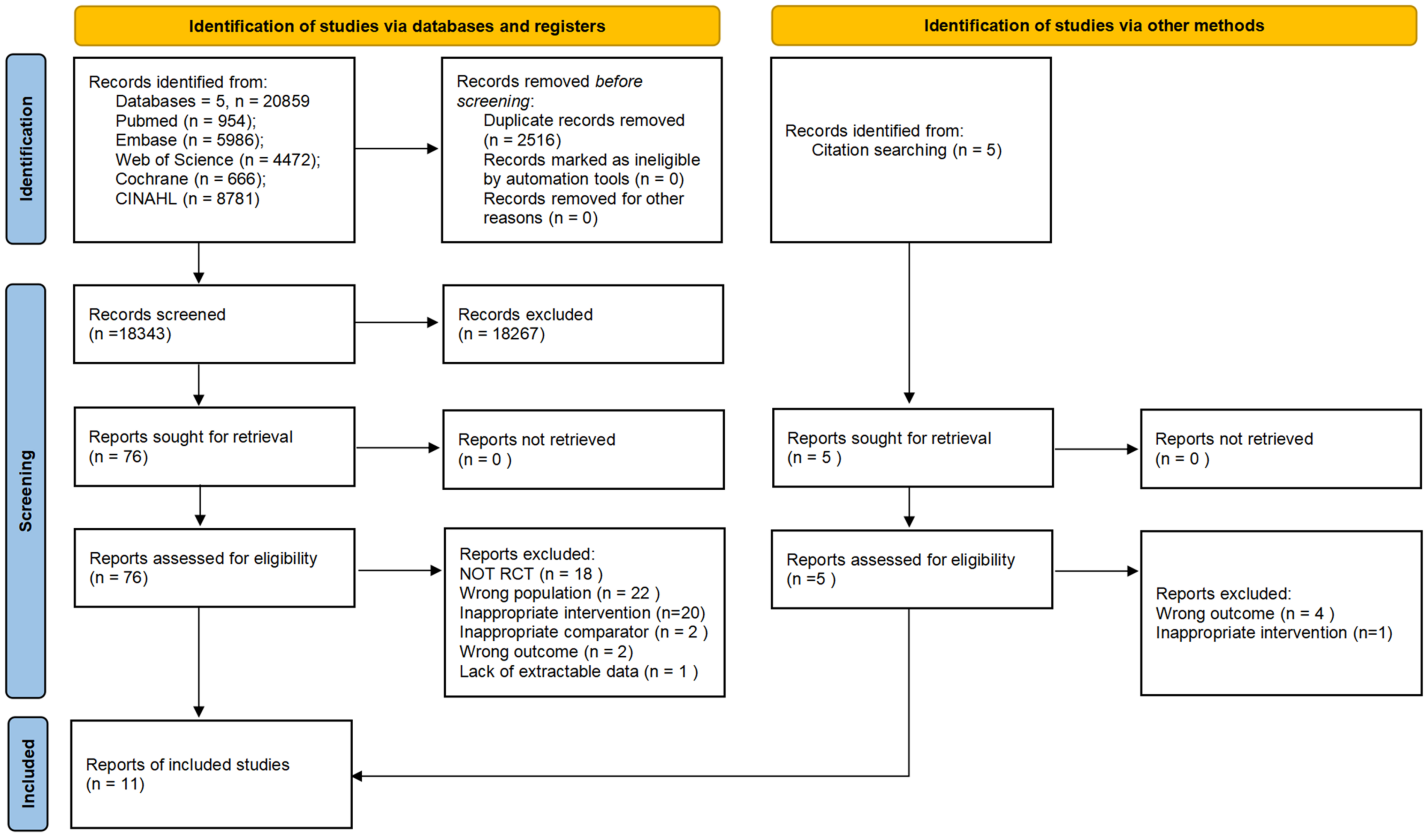
PRISMA flowchart.

Database searches initially identified 20,859 records, supplemented by 5 additional studies from citation searching. After removing duplicates (*n* = 2,516), 18,343 titles/abstracts were screened, with 18,267 excluded based on eligibility criteria. Subsequent full-text assessment of 81 studies yielded 11 studies meeting the inclusion criteria. The screening process is detailed in Figure 1.

### 3.2 Characteristics of the included studies

Our meta-analysis incorporated a total of 11 RCTs, involving 1,200 family members across six countries (USA, China, Germany, Netherlands, Switzerland, and Austria). The sample sizes ranged from 16 to 275. The detailed characteristics of the included studies are provided in Table 1.

**Table 1:** Characteristics of included studies.

Author (year) country	Age	Sample size	Intervention	Control	Outcomes (measurement)
Cox et al., 2018 (25) USA	I: 50 ± 14.9 C: 52.9 ± 15.2	I: 39 C: 47	Six weekly coping skills training telephone sessions	Education program with six informational videos	Anxiety (HADS-A), Depression (HADS-D), PTSD (IES-R)
Cox et al., 2019 (40) USA	I: 49.9 ± 13.5 C: 52.6 ± 11.6	I: 137 C: 138	Interactive web-based decision aid	Usual care	Anxiety (HADS-A), Depression (HADS-D), PTSD (PTSS)
Bannon et al., 2020 (36) USA	I: 56.7 ± 16.9 C: 51.7 ± 18.5	I: 7 C: 9	Recovering together: a six-session skills-based dyadic intervention	A three-page informational pamphlet	Anxiety (HADS-A), Depression (HADS-D), PTSD (PCL-C)
Gawlytta et al., 2022 (38) Germany	I: 55 (51–63) C: 53 (46–58)	I: 12 C: 13	Internet-based, therapist-led partner-assisted cognitive-behavioral writing therapy	Waitlist	PTSD (PCL-5)
Hoffmann et al., 2023 (39) Austria and Switzerland	47 ± 13	I: 46 C: 43	Intervention website with specific information about intensive care	Placebo website	Anxiety and Depression (HADS-Deutsch), PTSD (IES)
Petrinec et al., 2023 (24) USA	I: 55.4 ± 13.4 C: 58.5 ± 16.0	I: 30 C: 30	Mental Health App	No intervention	Anxiety (HADS-A), Depression (HADS-D), PTSD (PCL-5)
Yuan et al., 2023 (37) China	NR	I: 50 C: 48	Video visitation	Usual care	Anxiety (SAS)
Cox et al., 2024 (41) USA	I: 50 ± 13 C: 52 ± 16	I: 55 C: 56	ICUconnect (App)	Usual care	Anxiety (GAD-7), Depression (PHQ-9), PTSD (PTSS)
Cox et al., 2025 (42) USA	I: 57.3 ± 12.9 C: 57.4 ± 13.0	I: 76 C: 75	PCplanner (App)	Usual care	Anxiety (GAD-7), Depression (PHQ-9), PTSD (PTSS)
Drop et al., 2025 (26) Netherlands	I: 45 (20–69) C: 50 (24–72)	I: 100 C: 89	ICU-VR	Usual care	Anxiety (HADS-A), Depression (HADS-D), PTSD (IES-R)
Xiong et al., 2025 (23) China	NR	I: 50 C: 50	Wab-WPPEP intervention: a WeChat applet-based psychological empowerment program	Usual care	Anxiety (HADS-A), Depression (HADS-D), PTSD (IES-R)

I, intervention; C, control; NR, Not Reported; SDM, surrogate decision maker; ICU-VR, Intensive Care Unit-Specific Virtual Reality; APP, Application; PTSD, Post-Traumatic Stress Disorder; HADS, Hospital Anxiety and Depression Scale; HADS-A, Hospital Anxiety and Depression Scale-Anxiety; HADS-D, Hospital Anxiety and Depression Scale-Depression; IES, Impact of Event Scale; IES-R, Impact of Events Scale–Revised; PTSS, Posttraumatic Stress Syndrome Inventory; PCL-C, PTSD Checklist–Civilian Version; PCL-5, PTSD checklist for DSM-5; SAS, Chinese version of the Self-Rating Anxiety Scale; GAD-7, Generalized Anxiety Disorder 7 scale; PHQ-9: Patient Health Questionnaire-9 scale.

### 3.3 Characteristics of eHealth interventions

#### 3.3.1 Interaction modes and delivery format

Supplementary Table S5 summarizes the eHealth interventions. Throughout the trials, three studies adopted synchronous interaction, utilizing real-time communication via telephone calls or video conferencing (25, 36, 37). Three studies employed asynchronous interaction with delayed feedback (24, 38, 39). The remaining five studies implemented a hybrid approach, combining both synchronous and asynchronous methods to facilitate user interaction (23, 26, 40–42).

Four studies delivered app-based interventions (23, 24, 41, 42), and three studies employed web-based interventions (38–40). Two studies incorporated video conferencing (36, 37), while one study used a telephone-based intervention (25), and one study adopted virtual reality (VR) technology (26).

#### 3.3.2 Support strategies and initiation timing

Of the 11 studies included, five provided psychological support through various psychotherapies such as cognitive-behavioral therapy (CBT) and psychological empowerment (23–25, 36, 38). Three studies initiated psychological treatment during ICU hospitalization (23, 24, 36), while two initiated psychological treatment after ICU discharge (25, 38). The remaining six studies focused on information and communication support (26, 37, 39–42), delivering content related to ICU procedures, patient status updates, decision-making processes, rehabilitation, and palliative care. These interventions facilitated communication not only between ICU patients and family members but also between family members and ICU staff. Notably, all communication and information support eHealth interventions were initiated during ICU hospitalization.

#### 3.3.3 Duration and frequency of interventions

The duration and frequency of eHealth interventions varied across studies according to the type of support strategy. For psychological support-based eHealth interventions (*n* = 5), the prespecified duration was 5–8 weeks in most studies (4/5) (24, 25, 36, 38), whereas one study delivered the intervention from ICU admission to 1 month post-discharge (23). Regarding intervention frequency, three of five psychological support interventions specified a structured schedule of 1–2 sessions per week (25, 36, 38), while the remaining studies implemented either a daily program or a phase-based schedule (higher-intensity delivery during ICU stay followed by reduced frequency after discharge) (23, 24). For information and communication support-based eHealth interventions (*n* = 6), the intervention duration was ≤1 week or consisted of a single session delivery in most studies (4/6) (37, 40–42), the other two studies did not report a prespecified total intervention duration (26, 39). In terms of frequency, several information and communication interventions (4/6) (26, 39, 41, 42) did not prespecify a fixed dose and instead allowed self-paced, as-needed access to the platform or content during the intervention period and/or throughout follow-up, whereas the remaining two studies specified daily use or one-time exposure (37, 40).

#### 3.3.4 Intervention providers and user satisfaction

Across studies, eHealth interventions were delivered by multidisciplinary teams that variably included psychologists, palliative care specialists, physicians, nurses, and research staff. Provider composition differed by intervention content and format: psychotherapy-oriented interventions were typically led by professional psychologists (25, 36, 38), palliative care-focused interventions were co-delivered by ICU clinicians (physicians and/or nurses) and palliative care teams (42), and technology-mediated programs (websites/apps/VR) were commonly introduced by research staff with ICU clinicians supporting implementation (23, 26, 39). Notably, psychologists and palliative care teams were not routinely part of core ICU staffing.

Four of 11 studies reported family members’ satisfaction with the eHealth intervention (24, 26, 36, 37). Overall, satisfaction was favorable. In two studies (36, 37), satisfaction was significantly higher in the eHealth intervention group than in the control group, and families reported positive experiences with video-based modalities (video visits/videoconferencing). In the remaining two studies (24, 26), satisfaction was assessed only in the intervention arm; for example, in Petrinec et al. (24), satisfaction with a CBT-based mental health app averaged 4.19/5 (median 4; range 3–5). In Drop et al. (26), ICU-VR was highly rated: 90% of participants would recommend the intervention to other ICU families, 81% preferred VR over traditional informational brochures, 76% reported improved understanding of their relative’s ICU treatment, and 52% indicated that VR helped them process the ICU experience.

#### 3.3.5 Outcome measures

Among the 11 included studies, nine reported all three psychological outcomes in family members (anxiety, depression, and PTSD) (23–26, 36, 39–42), one study reported PTSD only (38), and one reported anxiety only (37). All psychological outcomes were assessed using self-report instruments, administered via paper-based or electronic questionnaires and/or through telephone interview.

### 3.4 Risk of bias

Supplementary Figure S1 shows the risk of bias for the 11 included studies. Overall, six studies were classified as having a high risk of bias (25, 26, 38–40, 42), while five studies were deemed to have some concerns (23, 24, 36, 37, 41). Regarding the randomization process, all studies provided details on the generation of random sequences, but one study raised concerns due to insufficient reporting of allocation concealment (24). In terms of deviations from the intended interventions, ten studies were rated as having some concerns (23–26, 36–38, 40–42), primarily due to the lack of blinding of participants and personnel. In the domain of missing outcome data, six studies were classified as having a high risk of bias (25, 26, 38–40, 42), as attrition rates exceeded 20% (33). All included studies had some concerns regarding outcome measurement, due to the reliance on self-reported measurements for assessing psychological symptoms. Finally, the risk of selective reporting was considered low across all studies.

### 3.5 Meta-analysis findings

#### 3.5.1 Anxiety symptoms

**Figure 2:**
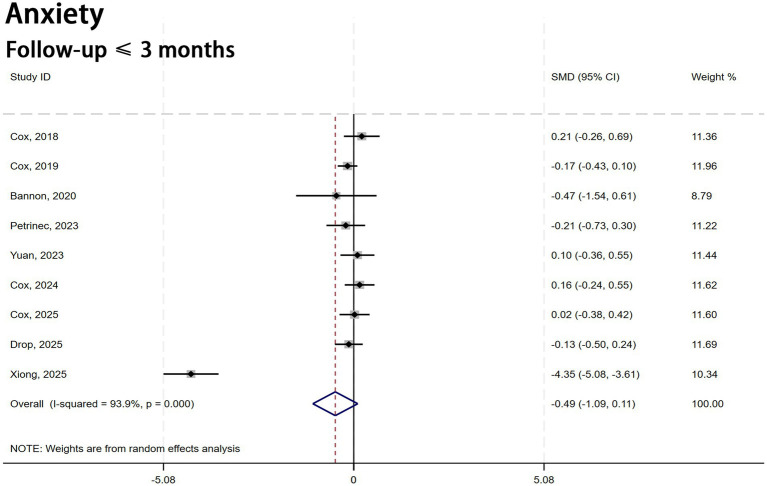
Forest plot of the effect of eHealth interventions on anxiety.

Nine studies involving 1,086 participants were included in the meta-analysis evaluating the effect of eHealth interventions on anxiety symptoms within a 3-month follow-up. The pooled results (Figure 2) indicated that eHealth interventions had no significant effect on anxiety symptoms among ICU patients’ families (SMD = −0.49, 95% CI: −1.09 to 0.11, *p* = 0.11, *I*^2^ = 93.9%).

#### 3.5.2 Depression symptoms

**Figure 3:**
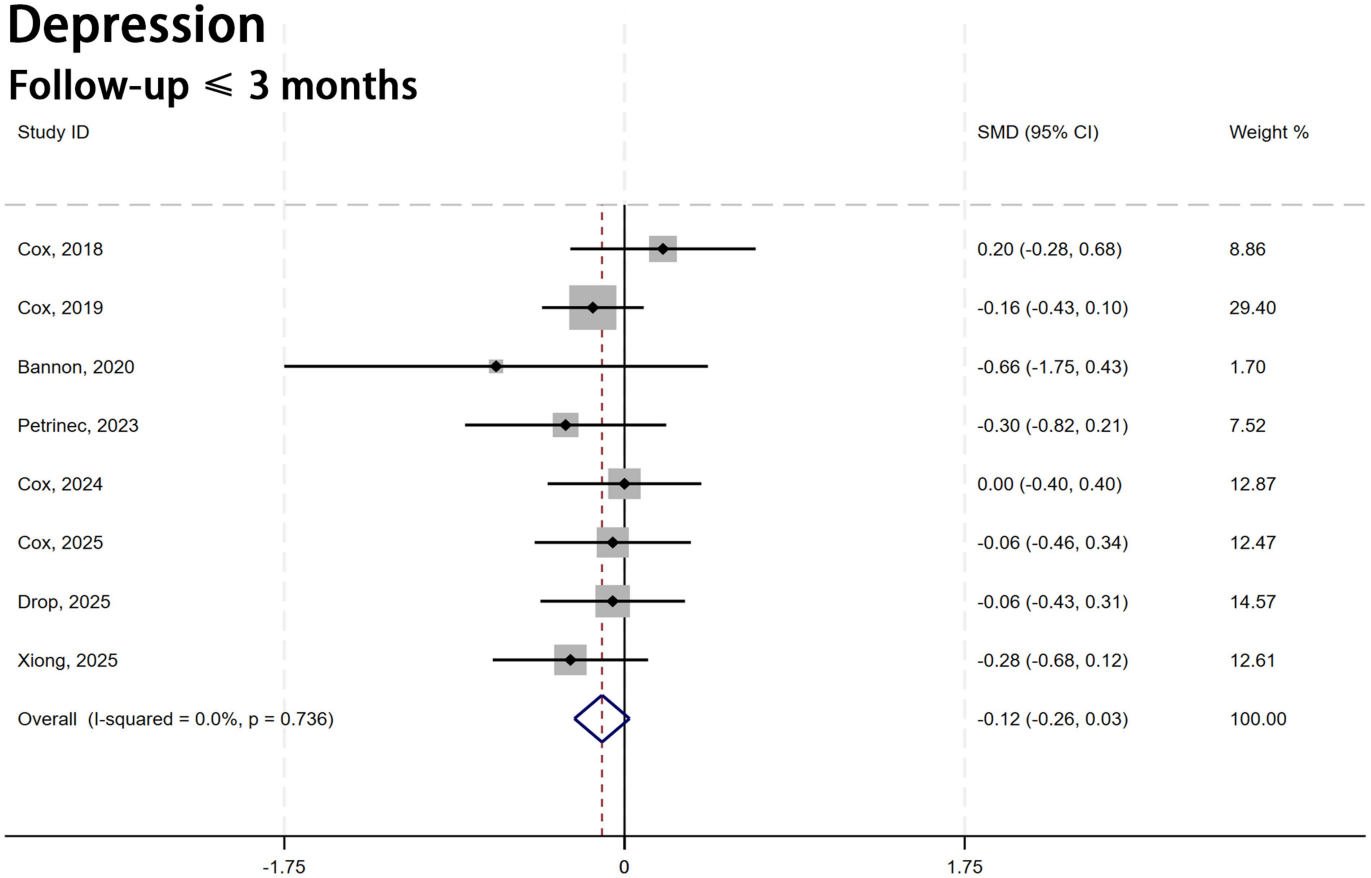
Forest plot of the effect of eHealth interventions on depression.

Eight studies, comprising 988 participants, examined depression symptoms. The pooled results (Figure 3) indicated that eHealth interventions had no significant effect on depression symptoms in the families of ICU patients (SMD = −0.12, 95% CI: −0.26 to 0.03, p = 0.11, *I*^2^ = 0.0%).

#### 3.5.3 PTSD symptoms

**Figure 4:**
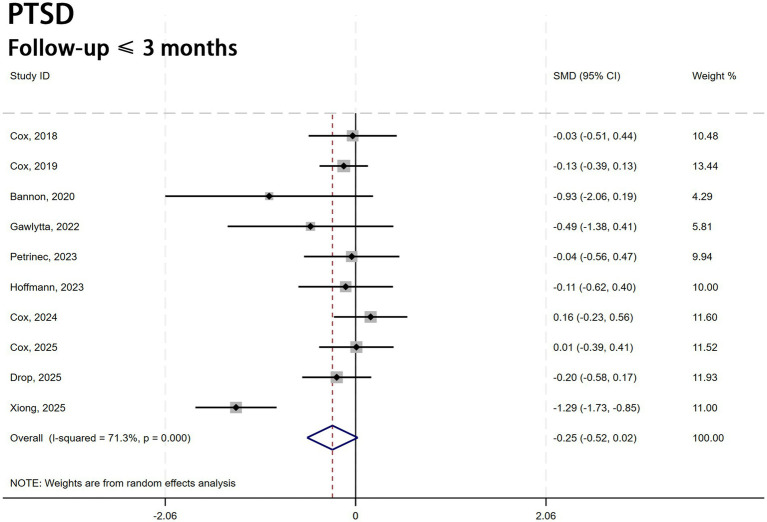
Forest plot of the effect of eHealth interventions on PTSD.

Ten studies, consisting of 1,102 participants, examined PTSD symptoms. The pooled results (Figure 4) demonstrated that eHealth interventions had no significant effect on PTSD symptoms in the families of ICU patients (SMD = −0.25, 95% CI: −0.52 to 0.02, *p* = 0.07, *I*^2^ = 71.3%).

### 3.6 Subgroup analysis

Table 2 presents the results of subgroup analyses, while the forest plots of the four subgroup analyses are provided in Supplementary Figures S2–S4. The results of the subgroup analyses indicated that eHealth interventions combined with early-stage psychological support led to a statistically significant improvement in depression symptoms among family members of ICU patients (SMD = −0.32, 95% CI: −0.62 to −0.01, *p* = 0.04, *I*^2^ = 0.0%). However, no significant effects were observed in other subgroups for any psychological outcomes among family members.

**Table 2:** Subgroup analyses of eHealth interventions on psychological outcomes.

Categorical covariate	Subgroup	No. of studies	Sample size	Outcome	*I*^2^	Overall effect (SMD) (95% CI)	Significance (*p*-value)
Interaction mode	Synchronous interaction	3	200	Anxiety	0.0%	0.10 (−0.21, 0.41)	0.53
		2	102	Depression	50.0%	0.06 (−0.38, 0.50)	0.79
		2	102	PTSD	52.5%	−0.33 (−1.17, 0.50)	0.43
	Asynchronous interaction	1	60	Anxiety	NA	−0.21 (−0.73, 0.30)	0.42
		1	60	Depression	NA	−0.30 (−0.82, 0.21)	0.25
		3	174	PTSD	0.0%	−0.13 (−0.47, 0.20)	0.44
	Hybrid interaction	5	826	Anxiety	96.8%	−0.84 (−1.82, 0.14)	0.09
		5	826	Depression	0.0%	−0.12 (−0.28, 0.04)	0.13
		5	826	PTSD	85.8%	−0.28 (−0.72, 0.15)	0.21
Support strategy	Psychological support	4	262	Anxiety	97.3%	−1.20 (−3.15, 0.76)	0.23
		4	262	Depression	16.7%	−0.17 (−0.42, 0.09)	0.20
		5	287	PTSD	79.8%	−0.53 (−1.14, 0.08)	0.09
	Communication and information support	5	824	Anxiety	0.0%	−0.04 (−0.20, 0.12)	0.58
		4	726	Depression	0.0%	−0.09 (−0.26, 0.08)	0.29
		5	815	PTSD	0.0%	−0.07 (−0.23, 0.09)	0.40
Initiation timing	Early-stage psychological support	3	176	Anxiety	97.6%	−1.68 (−4.47, 1.11)	0.24
		3	176	Depression	0.0%	−0.32 (−0.62, −0.01)	0.04*
		3	176	PTSD	84.8%	−0.74 (−1.66, 0.18)	0.11
	Late-stage psychological support	1	86	Anxiety	NA	0.21 (−0.26, 0.69)	0.38
		1	86	Depression	NA	0.20 (−0.28, 0.68)	0.42
		2	111	PTSD	0.0%	−0.13 (−0.55, 0.29)	0.54

NA: not applicable; *p < 0.05.

### 3.7 Sensitivity analyses and publication bias

The sensitivity analysis, conducted using the leave-one-out method, demonstrated that the effect size remained stable (Supplementary Figures S5–S7). Additionally, when studies with high attrition rates were excluded, the results showed no significant differences compared to the primary analysis. These findings further support the robustness of the pooled results (Supplementary Figure S8).

Egger’s test was performed to assess publication bias in studies examining the effects of eHealth interventions on PTSD symptoms within a 3-month follow-up. The results of Egger’s test (t = −0.77, *p* = 0.46) revealed no significant evidence of publication bias (Supplementary Figure S9).

## 4 Discussion

Based on the currently available evidence, our meta-analysis results indicated that eHealth interventions did not demonstrate statistically significant improvements in anxiety, depression, or PTSD symptoms among family members of ICU patients within 3 months of follow-up. Exploratory subgroup analyses suggested that eHealth interventions incorporating early-stage psychological support (initiated during ICU hospitalization) may be associated with improvements in depressive symptoms. In contrast, eHealth interventions primarily focused on information and communication support appear to have no effect on psychological outcomes. Similarly, eHealth interventions with different modes of interaction seem to have no effect on the psychological outcomes of ICU patients’ families. However, given the limited number of included studies and the substantial heterogeneity across interventions, these findings should be interpreted cautiously.

Conceptually, eHealth interventions are intended to extend care beyond the walls of the hospital and clinic (43), potentially increasing access to social support for family members of ICU patients and strengthening resilience to the detrimental effects of traumatic events (44). More specifically, eHealth interventions emphasizing information and communication support are generally designed to reduce informational and communication barriers between families and healthcare professionals by providing timely, structured information and facilitating communication, which may improve understanding and reduce stress (45). In contrast, eHealth interventions with psychological components aim to deliver accessible, evidence-based support (e.g., CBT or mindfulness) to reduce distress and promote psychological well-being (17, 46). Despite this theoretical promise and generally favorable satisfaction reported in a limited number of included studies (consistent with a prior review) (12), our pooled estimates did not demonstrate statistically significant short-term improvements in psychological outcomes. This discrepancy between acceptability and efficacy underscores the need to investigate why the anticipated benefits were not realized. Identifying the potential reasons for these null findings is therefore crucial for informing future intervention design.

There are several potential explanations for the lack of an overall effect of eHealth interventions on psychological outcomes of PICS-F. First, the digital delivery methods inherent in eHealth interventions may present limitations in establishing emotional connections. Although eHealth offers advantages in accessibility and continuity, family members of ICU patients, who face the fear of losing a loved one and the uncertainty of prognosis (16), may have heightened emotional support needs and therefore benefit more from intervention formats that better facilitate emotional connection (26). A previous systematic review by Cherak et al. (27) reported that predominantly face-to-face mental health interventions for families of critically ill patients were associated with reductions in anxiety and depressive symptoms, whereas the digitally delivered interventions in our review did not yield similar short-term benefits. Compared with face-to-face delivery, digital environments can attenuate the transmission of non-verbal cues and the expression of empathy and compassion (47, 48). Consistent with this, Kentish-Barnes et al. (49) highlighted the role of in-person communication skills, including non-verbal support and empathic responses, in improving anxiety, depression, and PTSD symptoms among ICU family members. In our review, even eHealth interventions using real-time communication for synchronous interaction struggled to fully replicate the quality of these face-to-face interactions, particularly when the strategy primarily emphasized the therapeutic information provision or communication facilitation. Taken together, rather than suggesting that eHealth lacks value, these findings may indicate that future eHealth interventions for populations with high emotional support needs could benefit from incorporating stronger empathic components and/or exploring blended models that combine digital delivery with targeted in-person contact.

Second, insufficient participant engagement in eHealth interventions may lead to lower-than-intended intervention exposure, thereby diluting potential effects. In practice, limited uptake has been reported in trials included in this review. Cox et al. (25) found that 53.8% of family members of ICU patients did not receive the planned telephone-based intervention due to the patient’s condition, being too busy, or lack of interest. Similarly, Petrinec et al. (24) reported low engagement with a mental health app, with only approximately one-third of participants using the application regularly over the 8-week period, while many logged in only once or a few times. This low engagement may relate to family members’ substantial caregiving burden, particularly during the post-ICU phase when caregiving responsibilities often increase (50–52). Since many eHealth programs extend into the post-ICU period, maintaining engagement amid high burden remains challenging. Stage-tailored eHealth interventions aligned with distinct phases of the ICU trajectory may be more acceptable, achieve higher intervention exposure, and potentially improve effectiveness (23, 26).

Third, the degree of personalization in eHealth interventions merits careful consideration, as intervention design should not be detached from the specific social context of family members of ICU patients (52). Previous research suggests that psychological outcomes among ICU family members are influenced by a wide range of factors that may confound intervention effects (53), including social determinants of health (SDOH) such as employment disruption, financial strain, caregiving burden, and post-discharge destination (discharge to home versus transfer to rehabilitation facilities) (8, 52, 54). These diverse social and caregiving circumstances give rise to highly heterogeneous needs among ICU family members, making it unlikely that a single, standardized intervention can effectively address psychological distress across this population (26). Previous evidence indicates that non-individualized, uniform approaches, such as standardized condolence letters, may even exacerbate adverse psychological outcomes (13), whereas interventions tailored to family members’ specific needs have shown beneficial effects (55). In this context, the studies included in our review encompassed both modular, personalized eHealth interventions and standardized eHealth interventions with predefined content; variability in the degree of personalization, together with unmeasured or insufficiently controlled confounding factors, may have obscured the overall effectiveness of eHealth interventions on PICS-F psychological outcomes.

Moreover, our subgroup analysis revealed that eHealth interventions offering early-stage (ICU hospitalization phase) psychological support may have a positive effect on depressive symptoms. This finding aligns with the perspective that treatment of PICS-F should likely start early (e.g., during the ICU stay of the family member and shortly after admission) instead of, “per definition,” start “post” a traumatic insult (1). It is further supported by Yoshihiro et al. (56), who raised concern that psychological interventions initiated at late-stage (post-ICU discharge) might not confer benefit and could potentially be associated with worse depression, PTSD, and anxiety in family members. Previous studies (57, 58), examining the trajectory of depressive symptoms among ICU patients’ family members have shown that, from ICU admission to post-discharge, their depressive symptoms either alleviate or persist over time, with the most severe phase occurring during the ICU stay. Targeted interventions based on the trajectory of psychological health changes in ICU patients’ family members were advisable (27). Families who serve as the primary caregivers during the ICU hospitalization and after discharge are exposed to prolonged caregiving stress, increasing the risk of depression recurrence and chronicity. Effective early intervention not only treats the current episode effectively but also has a prophylactic effect on future depression (59). Collectively, these findings highlight the critical importance of timing in introducing psychological support. Although the optimal duration and intensity of eHealth-based support remain uncertain due to substantial heterogeneity in intervention frequency and length across trials, available evidence suggests that initiating support during ICU admission holds promise. However, it must be acknowledged that this finding is preliminary and exploratory and may serve primarily as a basis for hypothesis generation. The potential beneficial effects of early-stage psychological support delivered through eHealth interventions on psychological outcomes among ICU patients’ families require further verification in future studies.

### 4.1 Limitations

Our findings should be interpreted within the context of the following limitations. First, substantial heterogeneity was observed across the included studies. The included eHealth interventions varied considerably in content and delivery format, which may have influenced the pooled estimates and obscured the true effects of eHealth interventions. Although subgroup and sensitivity analyses were conducted, the limited number of included studies restricted our ability to fully explore the sources of heterogeneity. Second, due to the high autonomy of participants in eHealth interventions, we were unable to verify the effective dosage, frequency, or duration of the interventions. Third, the overall certainty of the evidence was graded as very low according to the GRADE framework, as shown in Supplementary Table S6. Additionally, more than half of the included studies (6/11) exhibited high attrition rates, and the reasons for dropout may not be random. Participants with psychological dysfunction were more likely to withdraw from the studies, potentially leading to overestimation of eHealth intervention effects. Nevertheless, sensitivity analyses demonstrated that the effectiveness of eHealth interventions remained negative after excluding studies with high attrition.

These limitations underscore the need for further research that incorporates more rigorously designed interventions to generate more robust evidence on the effects of eHealth interventions.

## 5 Conclusion

Our study indicates that eHealth interventions did not demonstrate statistically significant short-term (≤3-month follow-up) improvements in anxiety, depression, or PTSD symptoms among family members of ICU patients. Preliminary findings suggest that eHealth interventions with early-stage psychological support may help alleviate depressive symptoms, although further rigorous verification is needed.

## Data Availability

All relevant data are within the manuscript and its Supplementary material.
