# Supplementary material for: Effects of eHealth interventions on psychological outcomes of post intensive care syndrome-family: a systematic review and meta-analysis
Source: Front Med (Lausanne). 2026 Jun 10;13:1847415. doi: 10.3389/fmed.2026.1847415 (PMC13290843; doi:10.3389/fmed.2026.1847415)
Supplement: Supplementary file 1 [file Data_sheet_1.docx]

**1. PRISMA 2020 Checklist**

**Table S1. Checklist**

|  | **Item #** | **Checklist item** | **Location** |
| --- | --- | --- | --- |
| **TITLE** | | |  |
| Title | 1 | Identify the report as a systematic review. | Manuscript-Title |
| **ABSTRACT** | | |  |
| Abstract | 2 | See the PRISMA 2020 for Abstracts checklist. | Manuscript-Abstract |
| **INTRODUCTION** | | |  |
| Rationale | 3 | Describe the rationale for the review in the context of existing knowledge. | Manuscript-Introduction |
| Objectives | 4 | Provide an explicit statement of the objective(s) or question(s) the review addresses. | Manuscript-Introduction |
| **METHODS** | | |  |
| Eligibility criteria | 5 | Specify the inclusion and exclusion criteria for the review and how studies were grouped for the syntheses. | Manuscript-Methods |
| Information sources | 6 | Specify all databases, registers, websites, organisations, reference lists and other sources searched or consulted to identify studies. Specify the date when each source was last searched or consulted. | Manuscript-Methods |
| Search strategy | 7 | Present the full search strategies for all databases, registers and websites, including any filters and limits used. | Supplementary-Table S2 |
| Selection process | 8 | Specify the methods used to decide whether a study met the inclusion criteria of the review, including how many reviewers screened each record and each report retrieved, whether they worked independently, and if applicable, details of automation tools used in the process. | Manuscript-Methods |
| Data collection process | 9 | Specify the methods used to collect data from reports, including how many reviewers collected data from each report, whether they worked independently, any processes for obtaining or confirming data from study investigators, and if applicable, details of automation tools used in the process. | Manuscript-Methods |
| Data items | 10a | List and define all outcomes for which data were sought. Specify whether all results that were compatible with each outcome domain in each study were sought (e.g. for all measures, time points, analyses), and if not, the methods used to decide which results to collect. | Manuscript-Methods |
|  | 10b | List and define all other variables for which data were sought (e.g. participant and intervention characteristics, funding sources). Describe any assumptions made about any missing or unclear information. | Manuscript-Methods |
| Study risk of bias assessment | 11 | Specify the methods used to assess risk of bias in the included studies, including details of the tool(s) used, how many reviewers assessed each study and whether they worked independently, and if applicable, details of automation tools used in the process. | Manuscript-Methods |
| Effect measures | 12 | Specify for each outcome the effect measure(s) (e.g. risk ratio, mean difference) used in the synthesis or presentation of results. | Manuscript-Methods |
| Synthesis methods | 13a | Describe the processes used to decide which studies were eligible for each synthesis (e.g. tabulating the study intervention characteristics and comparing against the planned groups for each synthesis (item #5)). | Manuscript-Methods |
|  | 13b | Describe any methods required to prepare the data for presentation or synthesis, such as handling of missing summary statistics, or data conversions. | Manuscript-Methods |
|  | 13c | Describe any methods used to tabulate or visually display results of individual studies and syntheses. | Manuscript-Methods |
|  | 13d | Describe any methods used to synthesize results and provide a rationale for the choice(s). If meta-analysis was performed, describe the model(s), method(s) to identify the presence and extent of statistical heterogeneity, and software package(s) used. | Manuscript-Methods |
|  | 13e | Describe any methods used to explore possible causes of heterogeneity among study results (e.g. subgroup analysis, meta-regression). | Manuscript-Methods |
|  | 13f | Describe any sensitivity analyses conducted to assess robustness of the synthesized results. | Manuscript-Methods |
| Reporting bias assessment | 14 | Describe any methods used to assess risk of bias due to missing results in a synthesis (arising from reporting biases). | Manuscript-Methods |
| Certainty assessment | 15 | Describe any methods used to assess certainty (or confidence) in the body of evidence for an outcome. | Manuscript-Methods |
| **RESULTS** | | |  |
| Study selection | 16a | Describe the results of the search and selection process, from the number of records identified in the search to the number of studies included in the review, ideally using a flow diagram. | Manuscript-Figure 1 |
|  | 16b | Cite studies that might appear to meet the inclusion criteria, but which were excluded, and explain why they were excluded. | Supplementary-Table S4 |
| Study characteristics | 17 | Cite each included study and present its characteristics. | Supplementary-Table S5 |
| Risk of bias in studies | 18 | Present assessments of risk of bias for each included study. | Manuscript-Results |
| Results of individual studies | 19 | For all outcomes, present, for each study: (a) summary statistics for each group (where appropriate) and (b) an effect estimate and its precision (e.g. confidence/credible interval), ideally using structured tables or plots. | Manuscript-Results |
| Results of syntheses | 20a | For each synthesis, briefly summarise the characteristics and risk of bias among contributing studies. | Manuscript-Results |
|  | 20b | Present results of all statistical syntheses conducted. If meta-analysis was done, present for each the summary estimate and its precision (e.g. confidence/credible interval) and measures of statistical heterogeneity. If comparing groups, describe the direction of the effect. | Manuscript-Results |
|  | 20c | Present results of all investigations of possible causes of heterogeneity among study results. | Manuscript-Results |
|  | 20d | Present results of all sensitivity analyses conducted to assess the robustness of the synthesized results. | Manuscript-Results |
| Reporting biases | 21 | Present assessments of risk of bias due to missing results (arising from reporting biases) for each synthesis assessed. | Manuscript-Results |
| Certainty of evidence | 22 | Present assessments of certainty (or confidence) in the body of evidence for each outcome assessed. | Supplementary-Table S6 |
| **DISCUSSION** | | |  |
| Discussion | 23a | Provide a general interpretation of the results in the context of other evidence. | Manuscript-Discussion |
|  | 23b | Discuss any limitations of the evidence included in the review. | Manuscript-Limitations |
|  | 23c | Discuss any limitations of the review processes used. | Manuscript-Limitations |
|  | 23d | Discuss implications of the results for practice, policy, and future research. | Manuscript-Discussion |
| **OTHER INFORMATION** | | |  |
| Registration and protocol | 24a | Provide registration information for the review, including register name and registration number, or state that the review was not registered. | Manuscript-Methods |
|  | 24b | Indicate where the review protocol can be accessed, or state that a protocol was not prepared. | Manuscript-Methods |
|  | 24c | Describe and explain any amendments to information provided at registration or in the protocol. | Manuscript-Methods |
| Support | 25 | Describe sources of financial or non-financial support for the review, and the role of the funders or sponsors in the review. | Manuscript-Statements |
| Competing interests | 26 | Declare any competing interests of review authors. | Manuscript-Statements |
| Availability of data, code and other materials | 27 | Report which of the following are publicly available and where they can be found: template data collection forms; data extracted from included studies; data used for all analyses; analytic code; any other materials used in the review. | Manuscript-Statements |

**2. Search strategy**

**Table S2. Search strategy**

| Pubmed  954 | #1 | (((((("post intensive care syndrome-family") OR ("post-intensive care syndrome family")) OR ("postintensive care syndrome-family")) OR ("postintensive care syndrome family")) OR ("PICS-F")) OR ("PICS-Family")) |
| --- | --- | --- |
|  | #2 | ((((((((((((family[MeSH Terms]) OR (spouses[MeSH Terms])) OR (caregivers[MeSH Terms])) OR (families)) OR ("family member")) OR (kinship)) OR (relatives)) OR (spouse)) OR ("domestic partner")) OR ("care giver")) OR (care*)) OR ("informal caregiver")) |
|  | #3 | ((((((intensive care units[MeSH Terms]) OR (Critical Care Nursing[MeSH Terms])) OR (Critical Care[MeSH Terms])) OR (Critical illness[MeSH Terms])) OR (icu)) OR ("intensive care")) |
|  | #4 | #2 AND #3 |
|  | #5 | #1 OR #4 |
|  | #6 | (((((((((((((Telemedicine[MeSH Terms]) OR (Virtual Reality[MeSH Terms])) OR (smartphone[MeSH Terms])) OR (telephone[MeSH Terms])) OR (computers[MeSH Terms])) OR (Mobile Applications[MeSH Terms])) OR (Videoconferencing[MeSH Terms])) OR (Digital Health[MeSH Terms])) OR (Telecommunications[MeSH Terms])) OR (Internet-Based Intervention[MeSH Terms])) OR (Internet[MeSH Terms])) OR (Software[MeSH Terms])) OR (Artificial Intelligence[MeSH Terms])) |
|  | #7 | ((((((((((ehealth) OR (mhealth)) OR ("mobile health")) OR ("health technology")) OR ("electronic health")) OR ("digital health")) OR (evisit)) OR ("e-visit")) OR (tele*)) OR ("Remote monitoring")) |
|  | #8 | ((((((((((((vr) OR (app)) OR (application)) OR ("wechat applet")) OR ("web-based")) OR ("web based")) OR ("internet-based")) OR ("internet based")) OR (cyber)) OR (online)) OR (AI)) OR ("Machine Intelligence")) |
|  | #9 | (((((((ipad) OR (phone)) OR (tablet)) OR (wearables)) OR ( sensors)) OR ("digital device")) OR ("electronic device")) |
|  | #10 | #6 OR #7 OR #8 OR #9 |
|  | #11 | (((((((anxiety[MeSH Terms]) OR (depression[MeSH Terms])) OR (Stress Disorders, Post-Traumatic[MeSH Terms])) OR (anxious*)) OR (depress*)) OR (ptsd)) OR ("Psychological outcomes")) |
|  | #12 | #5 AND #10 AND #11 |
|  | #13 | limit #12 to (yr="2012 - 2025") |
| Web of science 4472 | #1 | TS=("post intensive care syndrome-family" OR "post-intensive care syndrome family" OR "postintensive care syndrome-family" OR "postintensive care syndrome family" OR "PICS-F" OR "PICS-Family") |
|  | #2 | (TS=(family OR spouses OR caregivers) OR AB=(families OR "family member" OR kinship OR relatives OR spouse OR "domestic partner" OR "care giver" OR care* OR "informal caregiver")) |
|  | #3 | (TS=(intensive care units OR Critical Care Nursing OR Critical Care OR Critical illness) OR AB=(icu OR "intensive care")) |
|  | #4 | #2 AND #3 |
|  | #5 | #1 OR #4 |
|  | #6 | TS=(Telemedicine OR Virtual Reality OR smartphone OR telephone OR computers OR Mobile Applications OR Videoconferencing OR Digital Health OR Telecommunications OR Internet-Based Intervention OR Internet OR Software OR Artificial Intelligence) |
|  | #7 | AB=(ehealth OR mhealth OR "mobile health" OR "health technology" OR "electronic health" OR "digital health" OR evisit OR "e-visit" OR tele* OR "Remote monitoring") |
|  | #8 | AB=(vr OR app OR application OR "wechat applet" OR "web-based" OR "web based" OR "internet-based" OR "internet based" OR cyber OR online OR AI OR "Machine Intelligence") |
|  | #9 | AB=(ipad OR phone OR tablet OR wearables OR sensors OR "digital device" OR "electronic device") |
|  | #10 | #6 OR #7 OR #8 OR #9 |
|  | #11 | (TS=(anxiety OR depression OR "Stress Disorders, Post-Traumatic") OR AB=(anxious* OR depress* OR ptsd OR "Psychological outcomes")) |
|  | #12 | #5 AND #10 AND #11 |
|  | #13 | limit #12 to (yr="2012 - 2025") |
| EMBASE 5986 | #1 | post intensive care syndrome-family' OR 'post-intensive care syndrome family' OR 'postintensive care syndrome-family' OR 'postintensive care syndrome family' OR 'pics-f' OR 'pics-family' |
|  | #2 | family OR spouses OR caregivers OR families OR 'family member' OR kinship OR relatives OR spouse OR 'domestic partner' OR 'care giver' OR care* OR 'informal caregiver' |
|  | #3 | intensive care units' OR 'critical care nursing' OR 'critical care' OR 'critical illness' OR icu OR 'intensive care' |
|  | #4 | #2 AND #3 |
|  | #5 | #1 OR #4 |
|  | #6 | Telemedicine OR 'Virtual Reality' OR smartphone OR telephone OR computers OR 'Mobile Applications' OR Videoconferencing OR Digital Health OR Telecommunications OR 'Internet-Based Intervention' OR Internet OR Software OR 'Artificial Intelligence' |
|  | #7 | ehealth OR mhealth OR 'mobile health' OR 'health technology' OR 'electronic health' OR 'digital health' OR evisit OR 'e-visit' OR tele* OR 'Remote monitoring' |
|  | #8 | vr OR app OR application OR 'wechat applet' OR 'web-based' OR 'web based' OR 'internet-based' OR 'internet based' OR cyber OR online OR AI OR 'Machine Intelligence' |
|  | #9 | ipad OR phone OR tablet OR wearables OR sensors OR 'digital device' OR 'electronic device' |
|  | #10 | #6 OR #7 OR #8 OR #9 |
|  | #11 | anxiety OR depression OR 'stress disorders, post-traumatic' OR anxious* OR depress* OR ptsd OR "Psychological outcomes" |
|  | #12 | #5 AND #10 AND #11 |
|  | #13 | #12 AND [2012-2025]/py |
| CINAHL 8781 | S1 | (TX ("post intensive care syndrome-family" OR "post-intensive care syndrome family" OR "postintensive care syndrome-family" OR "postintensive care syndrome family" OR "PICS-F" OR "PICS-Family")) |
|  | S2 | (MH family) OR (TX (families OR "family member" OR kinship OR relatives)) |
|  | S3 | (MH spouses) OR (TX (spouse OR "domestic partner")) |
|  | S4 | (MH caregivers) OR (TX ("care giver" OR "informal caregiver")) |
|  | S5 | S2 OR S3 OR S4 |
|  | S6 | (MH intensive care units OR MH Critical Care Nursing OR MH Critical Care OR MH Critical illness) OR TX (icu OR "intensive care") |
|  | S7 | S5 AND S6 |
|  | S8 | S1 OR S7 |
|  | S9 | (MH Telemedicine) OR (MH "Virtual Reality") OR (MH smartphone) OR (MH telephone) OR (MH computers) OR (MH "Mobile Applications") OR (MH Videoconferencing) OR (MH "Digital Health") OR (MH Telecommunications) OR (MH "Internet-Based Intervention") OR (MH Internet) OR (MH Software) OR (MH "Artificial Intelligence") |
|  | S10 | (TX (ehealth OR mhealth OR "mobile health" OR "health technology" OR "electronic health" OR "digital health" OR evisit OR "e-visit" OR tele* OR "Remote monitoring")) |
|  | S11 | (TX (vr OR app OR application OR "wechat applet" OR "web-based" OR "web based" OR "internet-based" OR "internet based" OR cyber OR online OR AI OR "Machine Intelligence")) |
|  | S12 | (TX (ipad OR phone OR tablet OR wearables OR sensors OR "digital device" OR "electronic device")) |
|  | S13 | S9 OR S10 OR S11 OR S12 |
|  | S14 | (MH anxiety OR MH depression OR MH "Stress Disorders, Post-Traumatic") OR (TX (anxious* OR depress* OR ptsd OR "Psychological outcomes")) |
|  | S15 | S8 AND S13 AND S14 |
|  | S16 | limit S15 to (yr="2012 - 2025") |
| Cochrane  666 | #1 | post intensive care syndrome-family OR "post-intensive care syndrome family" OR "postintensive care syndrome-family" OR "postintensive care syndrome family" OR "PICS-F" OR "PICS-Family" |
|  | #2 | MeSH descriptor: [Family] explode all trees |
|  | #3 | MeSH descriptor: [Spouses] explode all trees |
|  | #4 | MeSH descriptor: [Caregivers] explode all trees |
|  | #5 | families OR "family member" OR kinship OR relatives OR spouse OR "domestic partner" OR "care giver" OR care* OR "informal caregiver" |
|  | #6 | #2 OR #3 OR #4 OR #5 |
|  | #7 | MeSH descriptor: [Intensive Care Units] explode all trees |
|  | #8 | MeSH descriptor: [Critical Care Nursing] explode all trees |
|  | #9 | MeSH descriptor: [Critical Care] explode all trees |
|  | #10 | MeSH descriptor: [Critical Illness] explode all trees |
|  | #11 | icu OR "intensive care" |
|  | #12 | #7 OR #8 OR #9 OR #10 OR #11 |
|  | #13 | #6 AND #12 |
|  | #14 | #1 OR #13 |
|  | #15 | MeSH descriptor: [Telemedicine] explode all trees |
|  | #16 | MeSH descriptor: [Virtual Reality] explode all trees |
|  | #17 | MeSH descriptor: [Smartphone] explode all trees |
|  | #18 | MeSH descriptor: [Telephone] explode all trees |
|  | #19 | MeSH descriptor: [Computers] explode all trees |
|  | #20 | MeSH descriptor: [Mobile Applications] explode all trees |
|  | #21 | MeSH descriptor: [Videoconferencing] explode all trees |
|  | #22 | MeSH descriptor: [Digital Health] explode all trees |
|  | #23 | MeSH descriptor: [Telecommunications] explode all trees |
|  | #24 | MeSH descriptor: [Internet-Based Intervention] explode all trees |
|  | #25 | MeSH descriptor: [Internet] explode all trees |
|  | #26 | MeSH descriptor: [Software] explode all trees |
|  | #27 | MeSH descriptor: [Artificial Intelligence] explode all trees |
|  | #28 | ehealth OR mhealth OR "mobile health" OR "health technology" OR "electronic health" OR "digital health" OR evisit OR "e-visit" OR tele* OR "Remote monitoring" |
|  | #29 | vr OR app OR application OR "wechat applet" OR "web-based" OR "web based" OR "internet-based" OR "internet based" OR cyber OR online OR AI OR "Machine Intelligence" |
|  | #30 | ipad OR phone OR tablet OR wearables OR sensors OR "digital device" OR "electronic device" |
|  | #31 | #15 OR #16 OR #17 OR #18 OR #19 OR #20 OR #21 OR #22 OR #23 OR #24 OR #25 OR #26 OR #27 OR #28 OR #29 OR #30 |
|  | #32 | MeSH descriptor: [Anxiety] explode all trees |
|  | #33 | MeSH descriptor: [Depression] explode all trees |
|  | #34 | MeSH descriptor: [Stress Disorders, Post-Traumatic] explode all trees |
|  | #35 | anxious* OR depress* OR ptsd OR "Psychological outcomes" |
|  | #36 | #32 OR #33 OR #34 OR #35 |
|  | #37 | #14 AND #31 AND #36 |
|  | #38 | limit #37 to (yr="2012 - 2025") |

**3. Eligibility criteria**

**Table S3. List of inclusion and exclusion criteria**

| **Criteria** | **Inclusion criteria** | **Exclusion criteria** |
| --- | --- | --- |
| **Population** | • Adult family members/informal caregivers (aged ≥ 18 years) of adult patients | • Families of pediatric, neonatal, or prenatal ICU patients |
|  | • ICU length of stay ≥24 hours |  |
|  |  |  |
| **Intervention** | • Deliver core content via electronic media | • Face-to-face/printed materials interventions |
|  | • With remote access | • No remote access |
|  |  | • Non-interactive prerecorded video/audio materials |
|  |  |  |
| **Comparison** | • Non-eHealth interventions (face-to-face/print-based) | • Control groups meeting eHealth intervention criteria |
|  | • Prerecorded non-interactive video/audio materials |  |
|  | • Usual care/placebo/waitlist/no intervention |  |
|  |  |  |
| **Outcomes** | • At least one psychological outcome (anxiety, depression, or PTSD) of ICU patients' family members was reported | • No psychological outcome (anxiety, depression, or PTSD) of ICU patients' family members was reported |
|  |  |  |
| **Type of design** | Randomized controlled trials |  |
| **Years of publication** | 2012 to 2025 |  |
| **Publication type** | Published trials |  |
| **Language** | No limit |  |

**4. Exclusion process**

**Table S4. List of full-text studies excluded with reasons (n=70)**

|  | **Author(year)** | **Name of the paper** | **Reasons for exclusion** | **Note** |
| --- | --- | --- | --- | --- |
| 1 | C.E.Cox, 2019 | Effects of mindfulness training programmes delivered by a self-directed mobile app and by telephone compared with an education programme for survivors of critical illness: a pilot randomised clinical trial | Wrong population |  |
| 2 | C.E.Cox, 2020 | Optimizing a self-directed mobile mindfulness intervention for improving cardiorespiratory failure survivors' psychological distress (LIFT2): design and rationale of a randomized factorial experimental clinical trial | Wrong population |  |
| 3 | H.Shahdosti, 2020 | Evaluating the effect of planned online video visitations on anxiety and depression of patients at open heart intensive care unit: A randomized controlled trial | Wrong population |  |
| 4 | J.H.Vlake, 2020 | Intensive Care specific Virtual Reality (ICU-VR) improves Post-Intensive Care Syndrome-related psychological sequelae in survivors of critical illness | Wrong population |  |
| 5 | G.Navarra-Ventura, 2021 | Virtual Reality-Based Early Neurocognitive Stimulation in Critically Ill Patients: a Pilot Randomized Clinical Trial | Wrong population |  |
| 6 | J.H.Vlake, 2021 | Virtual Reality to Improve Sequelae of the Postintensive Care Syndrome: A Multicenter, Randomized Controlled Feasibility Study | Wrong population |  |
| 7 | J.H.Vlake, 2021 | Intensive Care Unit-specific Virtual Reality for COVID-19 ICU survivors | Wrong population |  |
| 8 | C.Cuzco, 2022 | Impact of a Nurse-Driven Patient Empowerment Intervention on the Reduction in Patients' Anxiety and Depression During ICU Discharge: a Randomized Clinical Trial | Wrong population |  |
| 9 | V.Eleonora, 2022 | THE APPLICATION OF A BRIEF MINDFULNESS INTERVENTION IN PEOPLE WITH SEVERE COVID-19 | Wrong population |  |
| 10 | T.Jones, 2022 | (PO-128) Remote Virtual Reality Exposure Therapy for Post-Intensive Care Syndrome | Wrong population |  |
| 11 | J.H.Vlake, 2022 | Intensive Care Unit–Specific Virtual Reality for Critically Ill Patients with COVID-19: Multicenter Randomized Controlled Trial | Wrong population |  |
| 12 | B.Balakrishnan, 2023 | Effects of COVID-19 Acute Respiratory Distress Syndrome Intensive Care Unit Survivor Telemedicine Clinic on Patient Readmission, Pain Perception, and Self-Assessed Health Scores: Randomized, Prospective, Single-Center, Exploratory Study | Wrong population |  |
| 13 | C.E.Cox, 2022 | Feasibility of Mobile App-based Coping Skills Training for Cardiorespiratory Failure Survivors The Blueprint Pilot Randomized Controlled Trial | Wrong population |  |
| 14 | S.J.She, 2023 | Effect of CICARE communication nursing model combined with motivational psychological intervention in patients with post-intensive care unit syndrome | Wrong population |  |
| 15 | A.Stromberg, 2023 | Effects of Digital Yoga on Physical and Psychological Outcomes in Patients With Heart Failure: A Randomised Controlled Trial | Wrong population |  |
| 16 | C.E.Cox, 2024 | Mobile Mindfulness Intervention for Psychological Distress among Intensive Care Unit Survivors: A Randomized Clinical Trial | Wrong population |  |
| 17 | B.A.Khan, 2024 | Mobile Critical Care Recovery Program for Survivors of Acute Respiratory Failure: a Randomized Clinical Trial | Wrong population |  |
| 18 | B.Altay, 2025 | The Effects of Pulmonary Rehabilitation Applied to Intensive Care Unit Patients on Dyspnea, Quality of Life, Depression, Anxiety, and Daily Living Activities: a Randomized Controlled Study | Wrong population |  |
| 19 | A.Despoti, 2025 | Comparing virtual reality with traditional methods in cognitive rehabilitation in PICS syndrome | Wrong population |  |
| 20 | S.H.Khan, 2025 | Improving Recovery and Outcomes Every Day After the ICU (IMPROVE): a Randomized Controlled Trial | Wrong population |  |
| 21 | L.Murano, 2025 | The impact of Mindfulness-based stress reduction on Covid-19 survivors. A randomized controlled trial | Wrong population |  |
| 22 | B.A.Yilma, 2025 | The AI-Therapist Duo: Exploring the Potential of Human-AI Collaboration in Personalized Art Therapy for PICS Intervention | Wrong population |  |
| 23 | C. E. Cox,2012 | Development and preliminary evaluation of a telephone-based coping skills training intervention for survivors of acute lung injury and their informal caregivers | NOT RCT |  |
| 24 | M. K. Lee,2012 | The effects of video-based admission education on environmental stress, anxiety and nursing needs satisfaction among family member with patient in ICU | NOT RCT |  |
| 25 | C. E. Cox,2014 | Development and preliminary evaluation of a telephone-based mindfulness training intervention for survivors of critical illness | NOT RCT |  |
| 26 | M. Hoffmann,2019 | Online information for relatives of critically ill patients: Pilot test of the usability of an ICU families website | NOT RCT |  |
| 27 | M. Ziyaefard,2019 | Evaluation of the effects of social media-based training on satisfaction and anxiety among the families of patients at the intensive care unit after coronary artery bypass surgery | NOT RCT |  |
| 28 | K. Gorman,2020 | Successful implementation of a short message service (SMS) as intensive care to family communication tool | NOT RCT |  |
| 29 | E. E. Meyers,2020 | Building Resiliency in Dyads of Patients Admitted to the Neuroscience Intensive Care Unit and Their Family Caregivers: Lessons Learned From William and Laura | NOT RCT |  |
| 30 | A. Morgana,2021 | Psycological effects of video calls in caregivers of patients admitted in intensive care unit (ICU): A feasibility prospective pilot study | NOT RCT |  |
| 31 | A. Petrinec,2021 | Delivering Cognitive Behavioral Therapy for Post–Intensive Care Syndrome–Family via a Mobile Health App | NOT RCT |  |
| 32 | J. W. Shin,2021 | VidaTalk™ patient communication application "opened up" communication between nonvocal ICU patients and their family | NOT RCT |  |
| 33 | H. J. Yoo,2021 | The Effect of a Multifaceted Family Participation Program in an Adult Cardiovascular Surgery ICU | NOT RCT |  |
| 34 | C. Chen,2022 | COMFORT communication in the ICU: Pilot test of a nurse-led communication intervention for surrogates | NOT RCT |  |
| 35 | A. Kebapcı,2022 | The effect of structured virtual patient visits (sVPVs) on COVID‐19 patients and relatives' anxiety levels in intensive care unit | NOT RCT |  |
| 36 | M. M. Shirvani,2022 | The Effect of Telehealth Communication on Anxiety, Depression, and Visits of Family Members of COVID-19 Patients Admitted to Intensive Care Units | NOT RCT |  |
| 37 | N. P. Caballero-Suárez,2023 | Anxiety and Depression in Family Members of Critically Ill Covid-19 Inpatients: Brief Psychological Interventions via Telephone, an Exploratory Study | NOT RCT |  |
| 38 | T. d. S. R. Haack,2023 | Does an educational website improve psychological outcomes and satisfaction among family members of intensive care unit patients? | NOT RCT |  |
| 39 | K. A. Sarigiannis,2023 | Symptoms of Anxiety, Depression, and Stress among Families of Critically Ill Patients with COVID-19: A Longitudinal Clinical Trial | NOT RCT |  |
| 40 | A. Beutel,2025 | Patient perspectives on stress after ICU and a short primary care based psychological intervention - results from a qualitative sub‑study of the PICTURE trial | NOT RCT |  |
| 41 | C. E. Cox,2021 | Improving racial disparities in unmet palliative care needs among intensive care unit family members with a needs-targeted app intervention: the ICUconnect randomized clinical trial | Lack of extractable data |  |
| 42 | J. Zhou,2022 | Evaluation of the Application Effect of WeChat Platform-based Communication Mode in Family Members of Patients after Partial Pulmonary Resection in the Anesthesia Intensive Care Unit | Wrong outcome | The psychological outcomes (anxiety, depression, or PTSD) of ICU patients' family members were not reported. |
| 43 | H. Y. Woo,2024 | Efficacy of Virtual Visitation in ICU During COVID-19 Pandemic: The ICU Visits Randomized Controlled Trial | Wrong outcome | The psychological outcomes (anxiety, depression, or PTSD) of ICU patients' family members were not reported. |
| 44 | E. G. Lester, 2021 | Can a Dyadic Resiliency Program Improve Quality of Life in Cognitively Intact Dyads of Neuro-ICU Survivors and Informal Caregivers? Results from a Pilot RCT. | Wrong outcome | The psychological outcomes (anxiety, depression, or PTSD) of ICU patients' family members were not reported. |
| 45 | E. Shariati, 2021 | The effect of the web-based communication between a nurse and a family member on the perceived stress of the family member of patients with suspected or confirmed COVID-19: A parallel randomized clinical trial. | Wrong outcome | The psychological outcomes (anxiety, depression, or PTSD) of ICU patients' family members were not reported. |
| 46 | G. A. Pignatiello, 2019 | Comparing cognitive load levels among family members of the critically ill exposed to electronic decision aids. | Wrong outcome | The psychological outcomes (anxiety, depression, or PTSD) of ICU patients' family members were not reported. |
| 47 | A. O. Suen, 2021 | A pilot randomized trial of an interactive web-based tool to support surrogate decision makers in the intensive care unit. | Wrong outcome | The psychological outcomes (anxiety, depression, or PTSD) of ICU patients' family members were not reported. |
| 48 | J.W.Shin, 2025 | Preliminary efficacy of the vidatalkTM communication application on family psychological symptoms in the intensive care unit: A pilot study | Inappropriate comparator | The comparator group also met the inclusion criteria ( a standard tablet with MyChart Bedside and game apps) |
| 49 | A.M.Vranceanu,2020 | Feasibility and Efficacy of a Resiliency Intervention for the Prevention of Chronic Emotional Distress Among Survivor-Caregiver Dyads Admitted to the Neuroscience Intensive Care Unit: A Randomized Clinical Trial | Inappropriate comparator | The comparator group also met the inclusion criteria (six-session educational program: 2 at bedside and 4 via live video after discharge) |
| 50 | C.Jones,2012 | Intensive Care Diaries and Relatives' Symptoms of Posttraumatic Stress Disorder After Critical Illness: A Pilot Study | Inappropriate intervention | No electronic media were used. |
| 51 | D.B.White, 2012 | Nurse-led intervention to improve surrogate decision making for patients with advanced critical illness | Inappropriate intervention | No electronic media were used. |
| 52 | A.E.Barnato and R.M.Arnold, 2013 | The effect of emotion and physician communication behaviors on surrogates' life-sustaining treatment decisions: A randomized simulation experiment | Inappropriate intervention | No electronic media were used. |
| 53 | A.M.Torke, 2016 | The Family Navigator: a pilot intervention to support intensive care unit family surrogates | Inappropriate intervention | No electronic media were used. |
| 54 | V. C. L. Chiang, 2017 | Fulfilling the psychological and information need of the family members of critically ill patients using interactive mobile technology: A randomised controlled trial | Inappropriate intervention | The nurse-led electronic media use was confined to ICU wards without remote access capabilities. |
| 55 | M.Garrouste-Orgeas,2017 | The ICU-Diary study: prospective, multicenter comparative study of the impact of an ICU diary on the wellbeing of patients and families in French ICUs | Inappropriate intervention | No electronic media were used. |
| 56 | N.Kentish-Barnes, 2017 | Effect of a condolence letter on grief symptoms among relatives of patients who died in the ICU: a randomized clinical trial | Inappropriate intervention | No electronic media were used. |
| 57 | H. Soleimanpour,2017 | Psychological effects on patient's relatives regarding their presence during resuscitation | Inappropriate intervention | No electronic media were used. |
| 58 | J. L. McAdam and K. Puntillo,2018 | Pilot study assessing the impact of bereavement support on families of deceased intensive care unit patients | Inappropriate intervention | No electronic media were used. |
| 59 | P. L. Cairns,2019 | Stress Management Intervention to Prevent Post–Intensive Care Syndrome–Family in Patients' Spouses | Inappropriate intervention | No electronic media were used. |
| 60 | R. G. Rosa,2019 | Effect of Flexible Family Visitation on Delirium Among Patients in the Intensive Care Unit: The ICU Visits Randomized Clinical Trial | Inappropriate intervention | No electronic media were used. |
| 61 | A. Jaberi,2020 | Effect of family presence during teaching rounds on patient's anxiety and satisfaction in cardiac intensive care unit: A double-blind randomized controlled trial | Inappropriate intervention | No electronic media were used. |
| 62 | R. Rafeie,2021 | Effect of Couple Education on Spouses’ Anxiety and Treatment Adherence in Patients with Acute Coronary Syndrome Admitted to Cardiac Intensive Care Unit | Inappropriate intervention | No electronic media were used. |
| 63 | L. Showler,2022 | Communication with bereaved family members after death in the ICU: the CATHARTIC randomised clinical trial | Inappropriate intervention | No electronic media were used. |
| 64 | F. Zamani,2022 | The effect of family member's presence during teaching rounds on their anxiety in cardiac intensive care unit | Inappropriate intervention | No electronic media were used. |
| 65 | S. Ataeeara,2023 | The Effect of Transition Nursing Program from Intensive Care Units to General Units on Anxiety and Satisfaction of Patients and Their Families: A Clinical Trial Study | Inappropriate intervention | No electronic media were used. |
| 66 | K. Haines,2023 | Co-designed peer support to improve critical care recovery: icuRESOLVE Pilot randomised controlled trial | Inappropriate intervention | No electronic media were used. |
| 67 | B. M. Dijkstra,2024 | Effect of a Standardized Family Participation Program in the ICU: A Multicenter Stepped-Wedge Cluster Randomized Controlled Trial | Inappropriate intervention | No electronic media were used. |
| 68 | S. R. Guáqueta-Parada,2024 | Effectiveness of an intervention on the family's need for information in intensive care units | Inappropriate intervention | No electronic media were used. |
| 69 | E. Reifarth,2025 | Multicomponent Communication Intervention to Support Family Members of the Critically Ill: A Controlled Pre-Post Study | Inappropriate intervention | No electronic media were used. |
| 70 | R. A. Butler, 2025 | Randomized Clinical Trial of the Four Supports Intervention for Surrogate Decision-Makers in Intensive Care Units | Inappropriate intervention | No electronic media were used. |

**5. Characteristics of eHealth interventions**

**Table S5. Characteristics of eHealth interventions**

| Author (year) | Provider | Content | Frequency (Duration) | Delivery format | Interaction modes | Support strategy | Intervention initiation timing | Data collection time points (Attrition rate) |
| --- | --- | --- | --- | --- | --- | --- | --- | --- |
| Cox et al. (2018) | Psychologists; Study staff | The Coping Skills Training (CST) intervention, based on cognitive-behavioral therapy (CBT), includes the following components: 1) introduction and relaxation exercises; 2) progressive muscle relaxation; 3) pleasant activities and activity-rest cycles; 4) communication skills; 5) cognitive restructuring and pleasant imagery; and 6) a review session with planning for sustainability. Participants also gain access to a study website for further support. | Six weekly telephone sessions, each lasting approximately 30 minutes  (6-week intervention) | Telephone | Synchronous interaction | Psychological support | Post-discharge home period | Within 2weeks after arrival home, 3months after randomization (19.8%), and 6months after randomization (23.2%) |
| Cox et al. (2019) | NA | A web-based decision aid provided personalized prognostic estimates, explained treatment options, and interactively clarified patient values to inform a family meeting. | Single session on study Day 1 | Web | Hybrid  interaction | Communication and information support | ICU hospitalization period | Baseline, on study day3(10.5%), at 3months(17.8%) and 6months(20.4%) |
| Bannon et al. (2020) | Psychologists, nurses, neurointensivists and therapist | Recovering Together (RT) integrates mindfulness, CBT techniques, and positive psychology into a skills-based dyadic intervention. The program consists of six sessions and seven modules—two universal and five specific. The first two sessions are delivered to all dyads, teaching core skills such as mindfulness, dialectics, diaphragmatic breathing, and self-care. The following four sessions are tailored to address the unique needs of each dyad, focusing on specific challenges, sequelae, or concerns identified collaboratively by the therapist and the dyad, from the five available modules. | One session one week; 2 sessions at bedside and 4 sessions via live video after discharge (6-week intervention) | Live video | Synchronous interaction | Psychological support | ICU hospitalization period | Baseline, posttreatment(6.3%) and 3months(12.5%) |
| Gawlytta et al. (2022) | Therapist | iCBT: internet-based, therapist-led partner-assisted cognitive-behavioural writing therapy. After completion of each assignment, the therapist provided individual feedback and further writing instructions to the participant within one workday. | 10 writing assignments over a 5-week period; two 50 min internet-based writing assignments per week (5-week intervention) | Internet-based web portal | Asynchronous interaction | Psychological support | Post-discharge home period | Baseline, posttreatment(20%) |
| Hoffmann et al. (2023) | NA | Intervention website: It contained basic information about the participating ICUs, a chat with ICU experts (forum), videos, detailed descriptions of different ICU topics, stories of critically ill patients and their families, information on stress and anxiety reduction, resources for children, including videos and a picture story, and a glossary. | NA | Website | Asynchronous interaction | Communication and information support | ICU hospitalization period | Baseline, day 30(16.9%), day 90(33.7%), day365(46.1%) |
| Petrinec et al. (2023) | NA | The Mental Health App offers digital CBT interventions, beginning with the "Feeling Better" module, followed by additional modules: "Taking Control," "Building Confidence," and "Mindfulness." Each module consists of 6 to 9 lessons, with each lesson taking approximately 15 minutes to complete. | one lesson per day (8-week intervention) | App | Asynchronous interaction | Psychological support | ICU hospitalization period | At enrollment, day30(0%), day 60(3.3%) |
| Yuan et al. (2023) | ICU nurses | Video visitation: A video call method for communication among patients, family members, and the patient’s nurse, along with the routine visit method used in the control group. A research nurse was responsible for initiating a WeChat video call with the patients' family members every afternoon using a mobile device. The nurse would also be at the patient’s bedside to ensure their safety during the intervention. | once daily during ICU hospitalization  (Average length of ICU stay: 3.8 days) | Video conferencing via WeChat | Synchronous interaction | Communication and information support | ICU hospitalization period | Admission into and discharge from the ICU(0%) |
| Cox et al. (2024) | Physicians | ICUconnect: A mobile application that displayed family-reported needs over time and provided ICU attending physicians with automated timeline-driven communication advice on how to address individual needs. | NA  (7- to 10-day intervention) | App | Hybrid  interaction | Communication and information support | ICU hospitalization period | Baseline, day1(0%), day3(0.9%), day7(5.4%), 3months(11.7%) |
| Cox et al. (2025) | Physicians and palliative care specialists | PCplanner: An automated electronic health，record–integrated, mobile application–based communication platform that displayed family-reported needs, coached ICU attending physicians on addressing needs, and prompted palliative care consultation with the palliative care team to provide collaborative palliative care. | NA  (7-day intervention) | App | Hybrid  interaction | Communication and information support | ICU hospitalization period | Baseline, day3(15.2%), day7(24.5%), 3months(37.1%) |
| Drop et al. (2025) | trained ICU researchers | VR intervention: A VR session (14 min) during the first ICU visit using a head-mounted VR device and cardboard VR glasses, with a provided access link. The information provided includes: 1) an introduction by an intensivist and an ICU nurse to welcome the relative to the ICU and VR environment, explaining daily movements in the ICU; 2) an explanation of monitors and noises in the ICU room; 3) information regarding mechanical ventilation, intubation, and tracheal tube suction; 4) information on the necessity of central/peripheral lines and intravenous drips; 5) information on the necessity of the treatment team and ICU workflow. | NA | VR | Hybrid  interaction | Communication and information support | ICU hospitalization period | Baseline, at ICU discharge(40.7%) , at 1month(41.8%) , 3months(40.2%), and 6months(45.0%) post-ICU discharge |
| Xiong et al. (2025) | Physicians, nurses and nursing master's students | Wab-WPPEP intervention: a WeChat applet-based psychological empowerment program for family caregivers. It provided emotional support (remote video visits, daily emotional interactions, and message exchanges), professional support ( information on the ICU work environment, heart valve disease, postoperative care, and rehabilitation), and appraisal support (expert consultations: family caregivers can leave messages in the WeChat applets, and medical professionals will respond at designated times each day ). | Delivery of relevant content:(a) ICU period: once daily; (b) General ward period: twice weekly; (c) Post-discharge period: once weekly. (from ICU admission to one month post-discharge) | WeChat applet | Hybrid interaction | Psychological support | ICU hospitalization period | Baseline, before transfer from the ICU to the general ward(1%), before discharge from the hospital(1%), and 1month after discharge (3%) |
| Abbreviations: NA, Not Applicable; CBT, cognitive-behavioral therapy; App, Application; VR, Virtual Reality; | | | | | | | |  |

**6. Risk of bias**


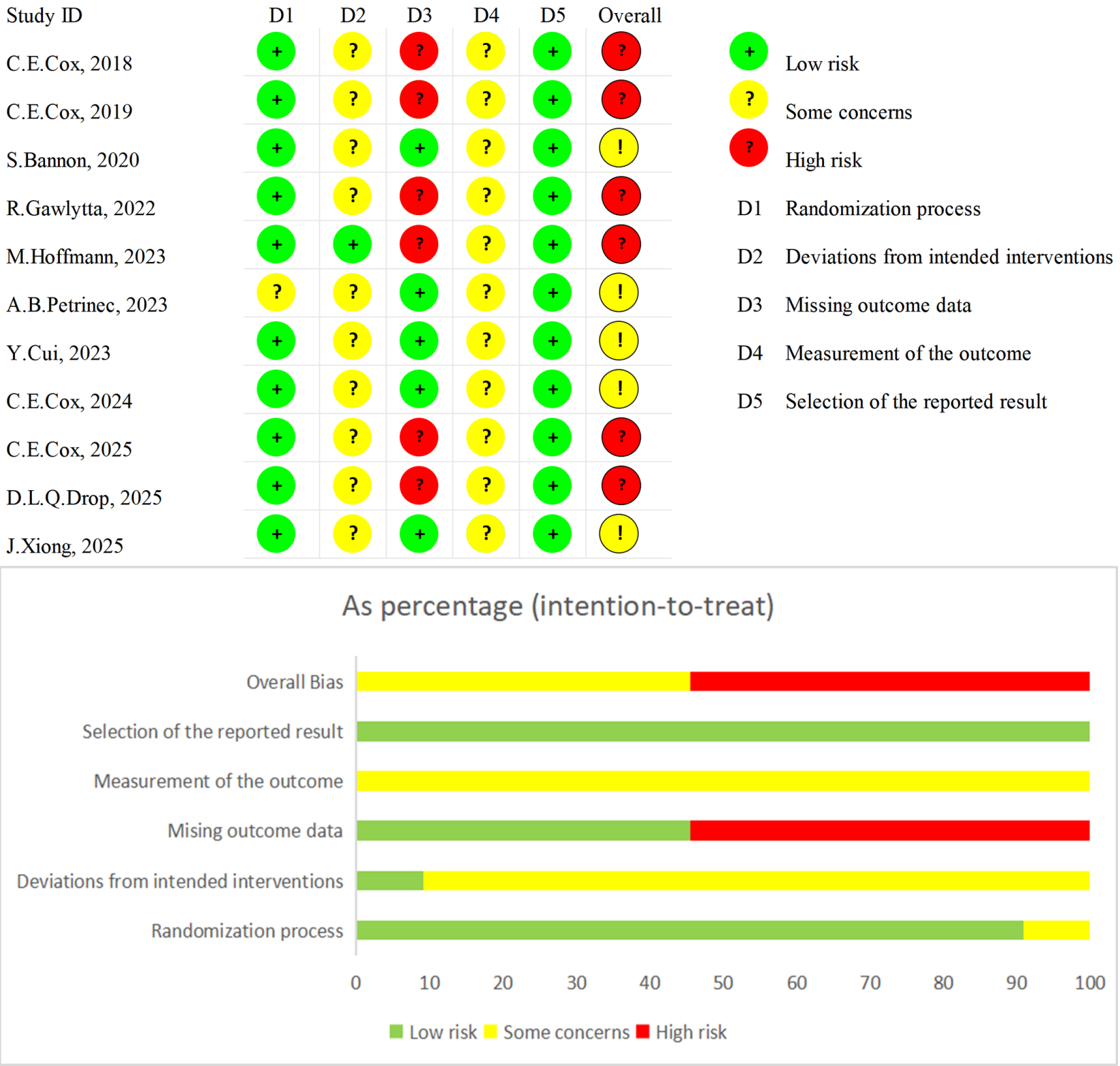


Fig. S1. Risk of bias

**7. GRADE**

**Table S6. GRADE evidence profile**

| Quality of evidence factors | | | | | | | | No of patients | Effect | | | Certainty of evidence | Importance |
| --- | --- | --- | --- | --- | --- | --- | --- | --- | --- | --- | --- | --- | --- |
| No of studies | Study design | Risk of bias | Inconsistency | Indirectness | Imprecision | Publication bias | Other considerations |  | Effect size | 95% CI | I^2^ |  |  |
| **Anxiety symptoms** | | | | | | | | | | | | | |
| **Follow-up ≤ 3 months** | | | | | | | | | | | | | |
| 9 | RCTs | Serious^1^ | Very serious^3^ | Serious^4^ | Very serious^6^ | NA | None | 1086 | SMD = -0.49 | (-1.09, 0.11) | 93.9% | Very low ⨁◯◯◯ | Critical |
| **Depression symptoms** | | | | | | | | | | | | | |
| **Follow-up ≤ 3 months** | | | | | | | | | | | | | |
| 8 | RCTs | Serious^1^ | Not serious | Serious^4^ | Serious^5^ | NA | None | 988 | SMD = -0.12 | (-0.26, 0.03) | 0% | Very low ⨁◯◯◯ | Critical |
| **PTSD symptoms** | | | | | | | | | | | | | |
| **Follow-up ≤ 3 months** | | | | | | | | | | | | | |
| 10 | RCTs | Serious^1^ | Serious^2^ | Serious^4^ | Very serious^6^ | Not detected | None | 1102 | SMD = -0.25 | (-0.52, 0.02) | 71.3% | Very low ⨁◯◯◯ | Critical |

^1^due to nearly 1/2 from middle or high risk

^2^due to 50%<I²<75%

^3^due to I²≥75%

^4^doe to different comparator and intervention timing

Thresholds were adopted as small (SMD = ±0.2), moderate (SMD = ±0.5), and large effects (SMD = ±0.8) (Schünemann et al., 2022)

^5^due to 1 threshold is crossed

^6^due to ≥2 thresholds are crossed

**8. Subgroup analyses**

**8.1. Subgroup analysis based on interaction mode**


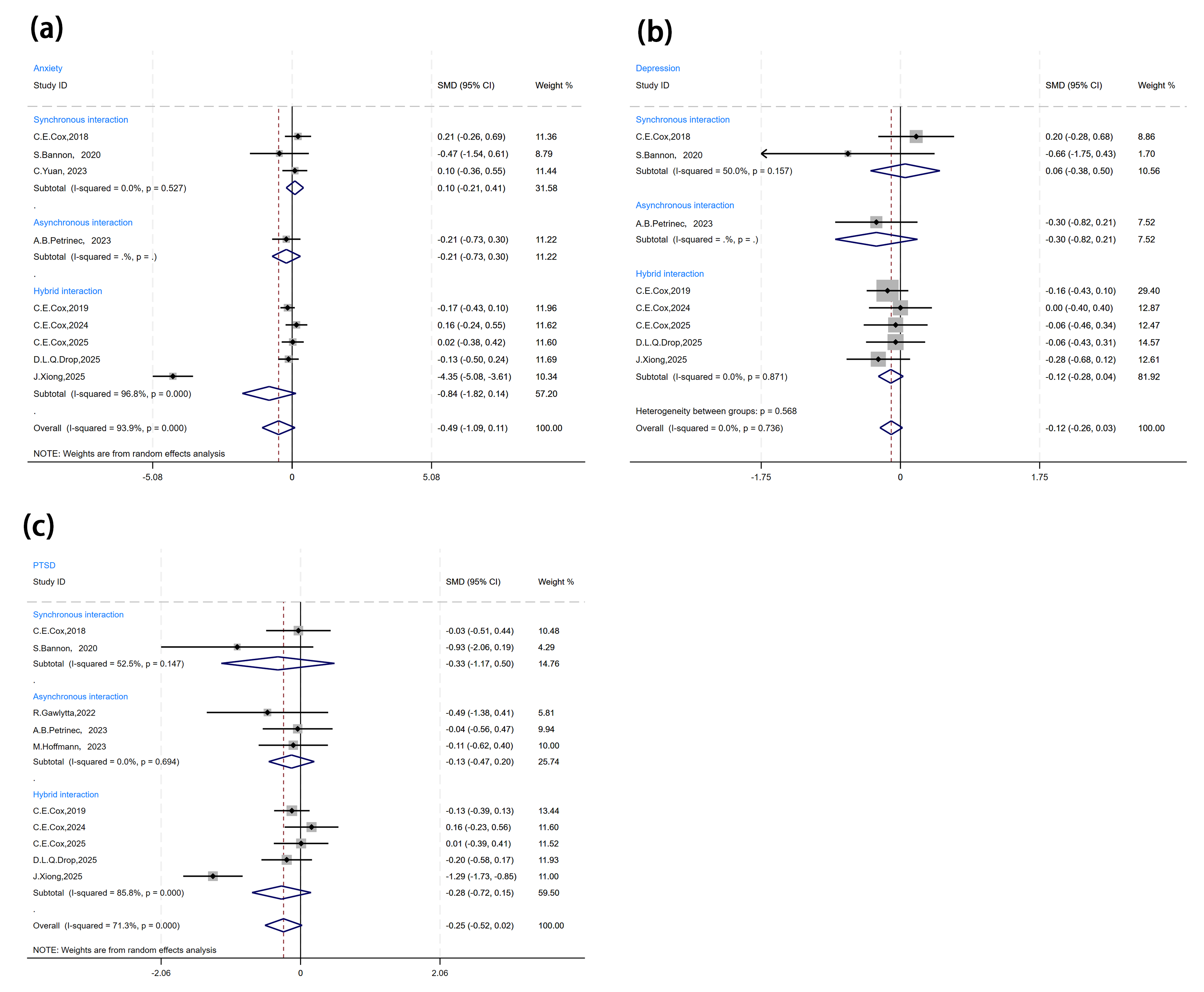


Fig. S2. Subgroup analysis of eHealth interventions on (a) anxiety, (b) depression, and (c) PTSD symptoms by interaction mode

**8.2. Subgroup analysis based on support strategy**


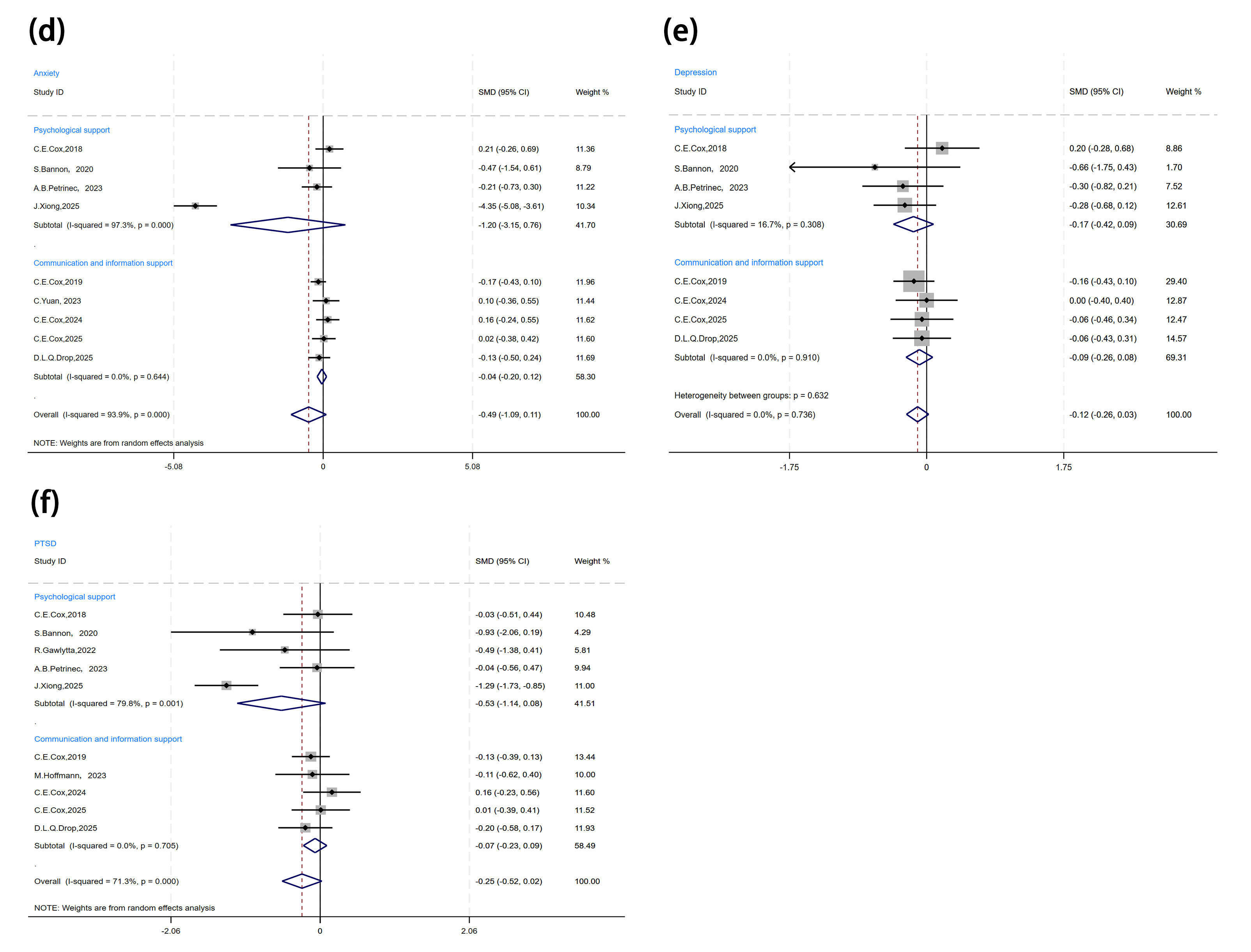


Fig. S3. Subgroup analysis of eHealth interventions on (d) anxiety, (e) depression, and (f) PTSD symptoms by support strategy

**8.3. Subgroup analysis based on initiation timing**


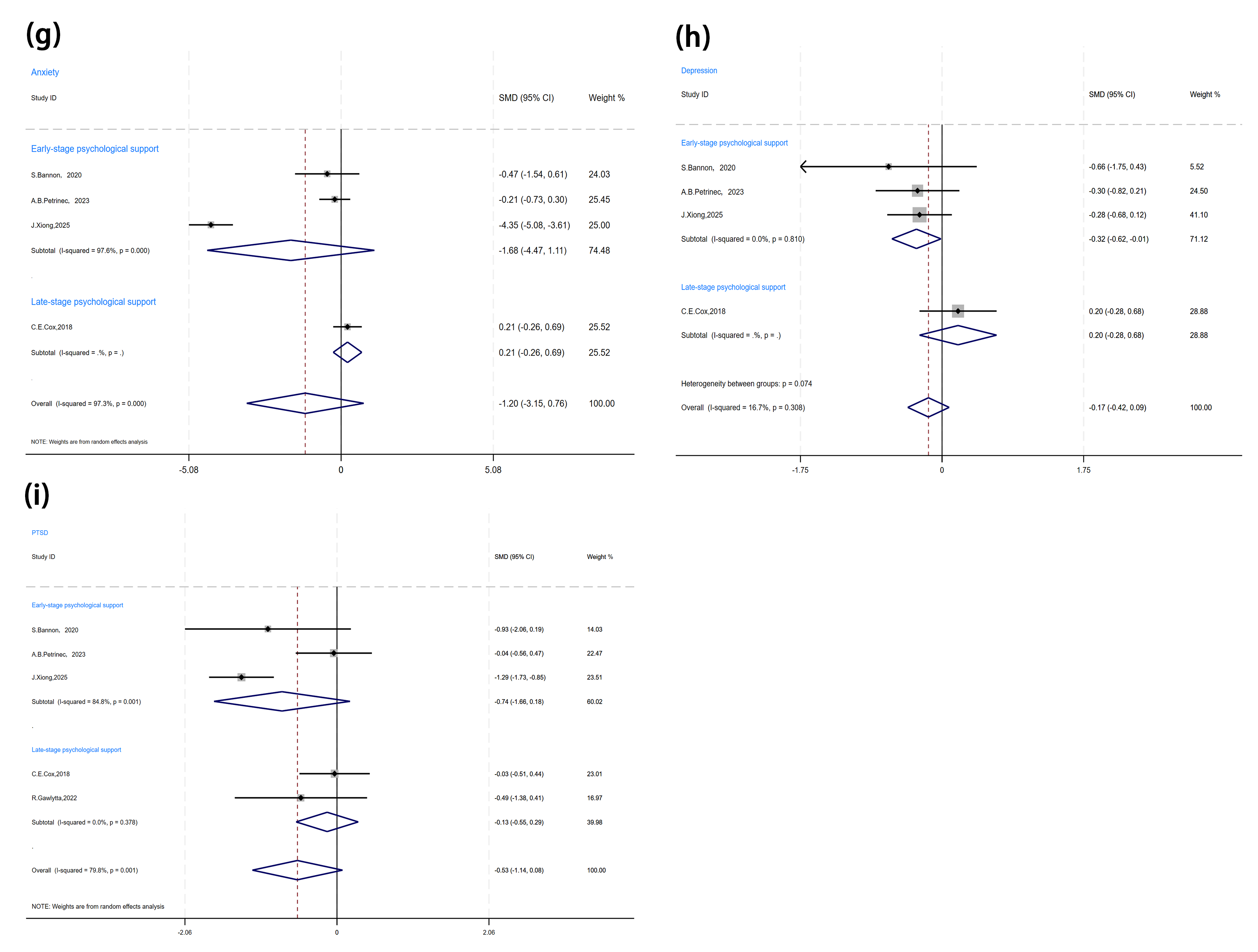


Fig. S4. Subgroup analysis of eHealth interventions on (g) anxiety, (h) depression, and (i) PTSD symptoms by initiation timing

**9. Sensitivity analysis**

**9.1 Sensitivity analysis using the leave-one-out method**


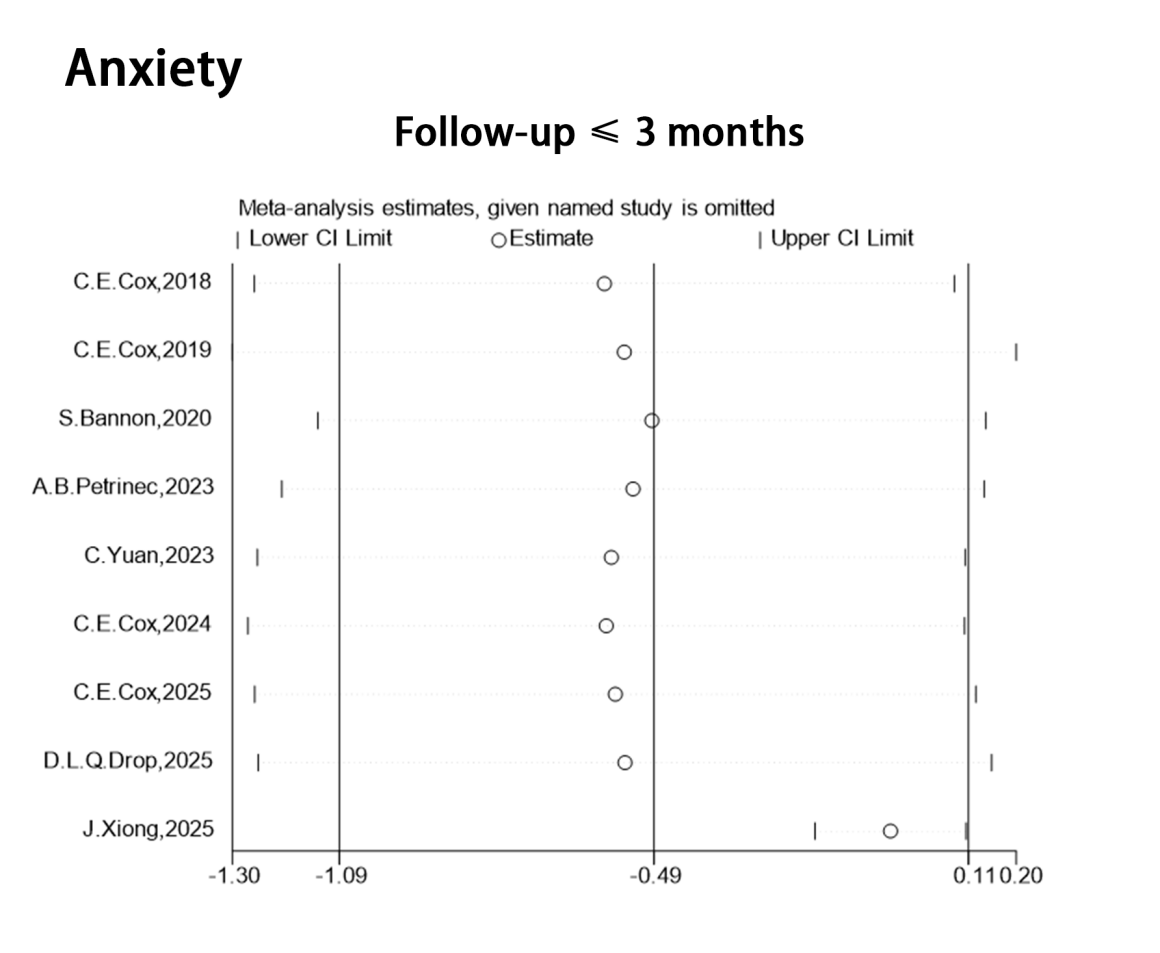


Fig. S5. Sensitivity analysis of eHealth interventions on anxiety symptoms within 3 months follow-up


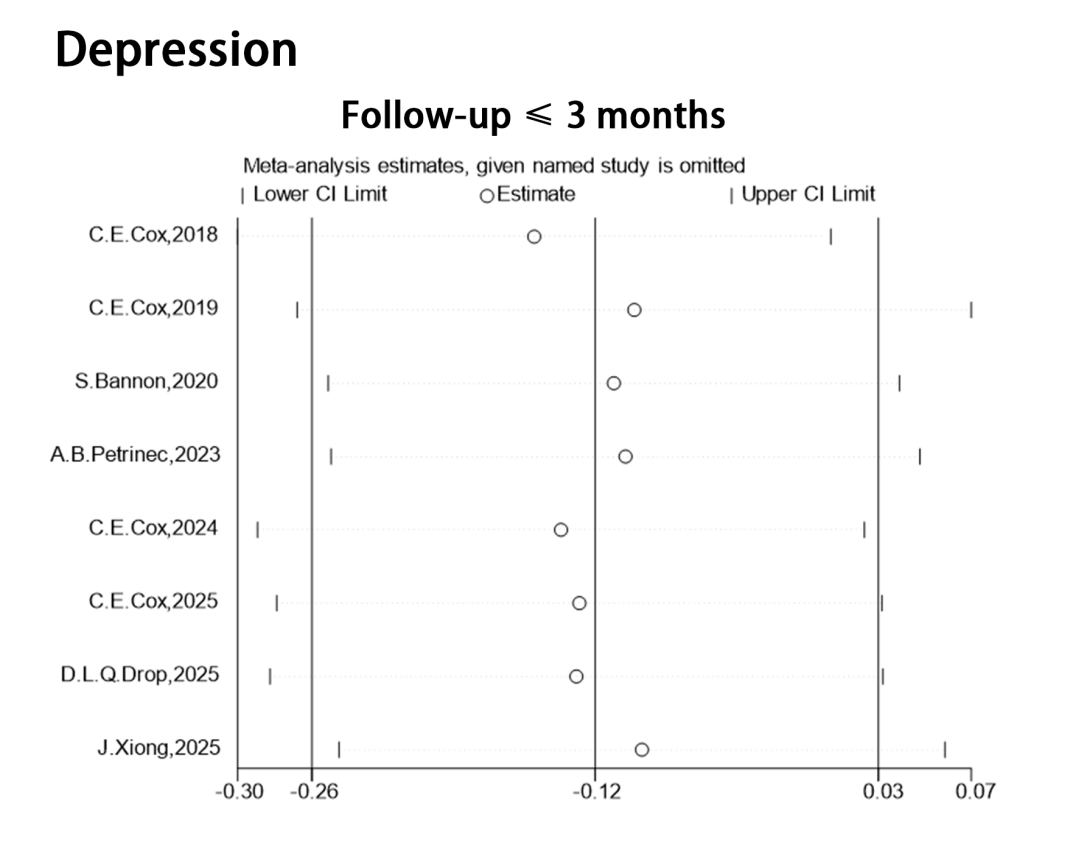


Fig. S6. Sensitivity analysis of eHealth interventions on depression symptoms within 3 months follow-up


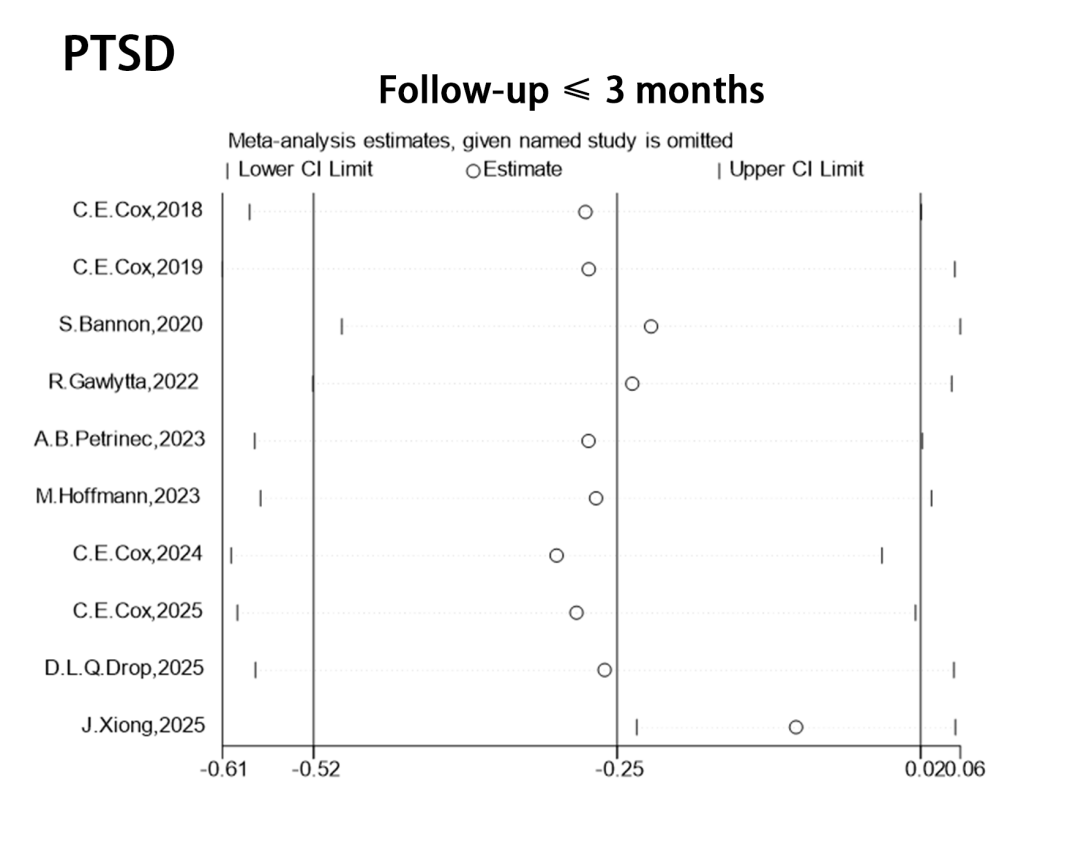


Fig. S7. Sensitivity analysis of eHealth interventions on PTSD symptoms within 3 months follow-up

**9.2 Sensitivity analysis by excluding studies with high attrition rates (≥20%)**


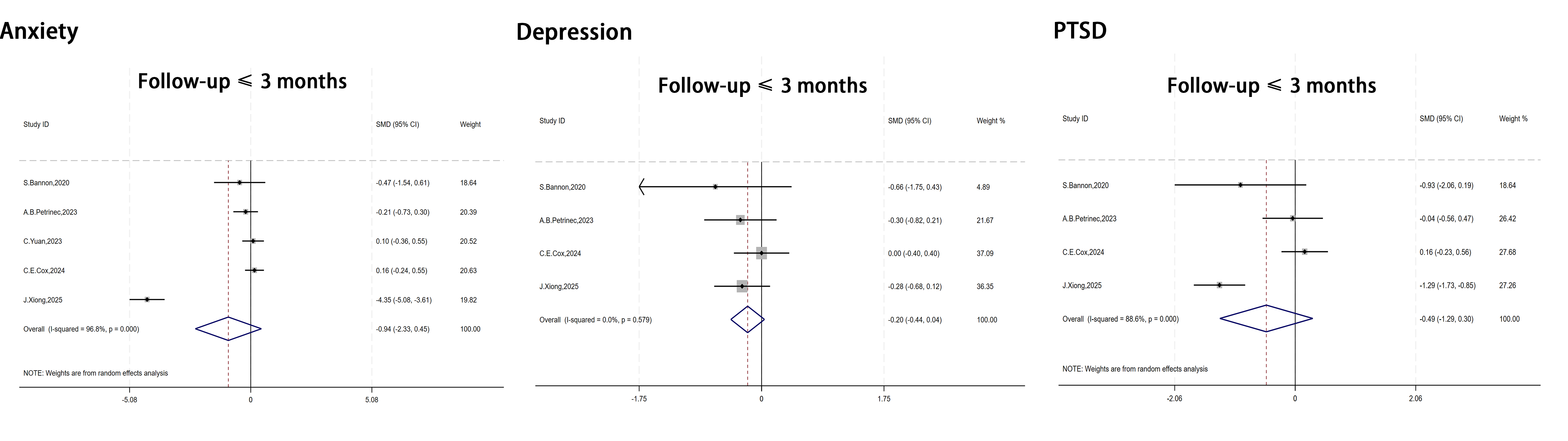


Fig. S8. Sensitivity analysis of eHealth interventions on anxiety, depression, and PTSD symptoms within 3 months follow-up, excluding studies with high attrition rates (≥20%)

**10. Publication bias**


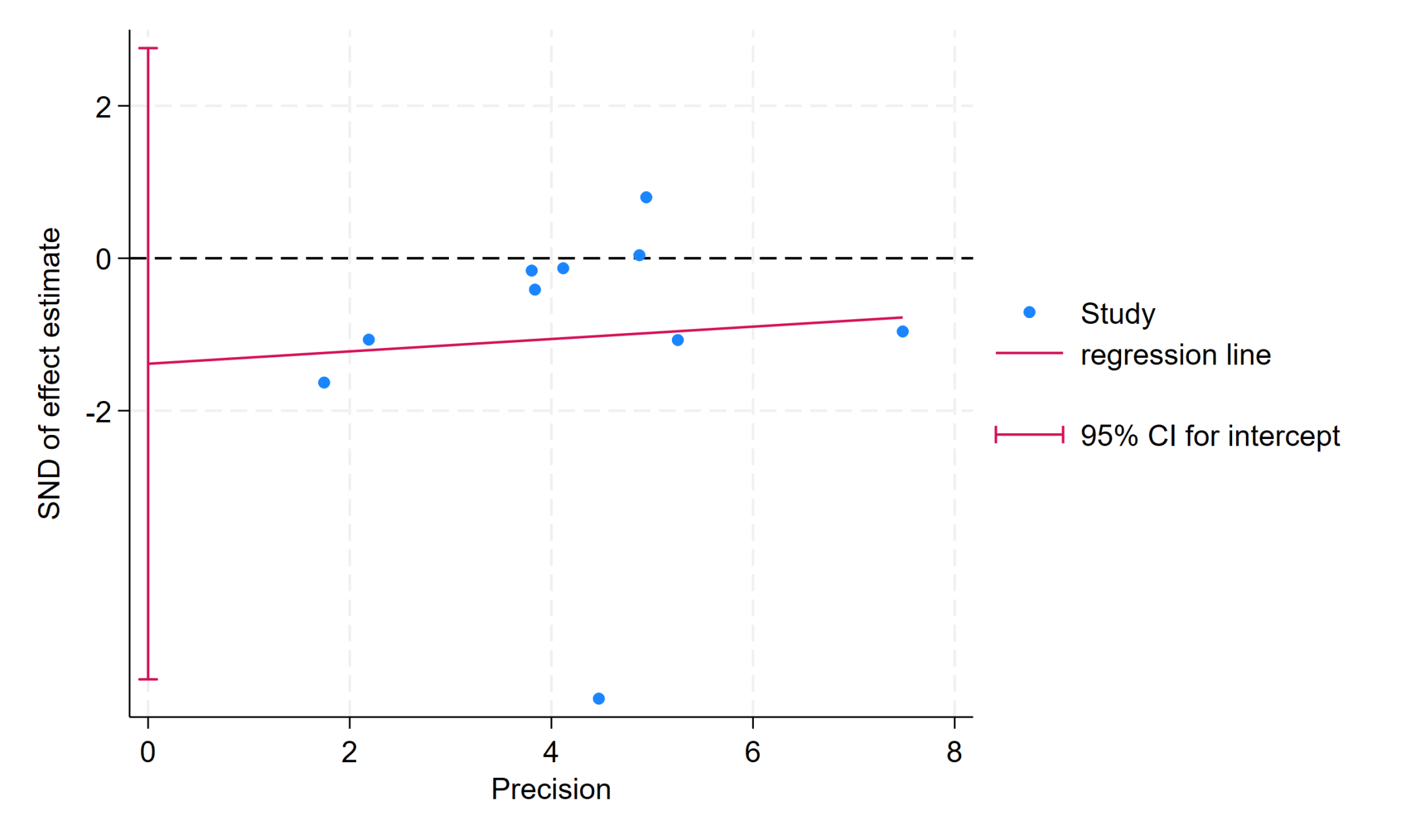


Fig. S9. Egger's regression plot of eHealth intervention effects on PTSD symptoms within 3-month follow-up

**References：**

Schünemann, H.J., Neumann, I., Hultcrantz, M., Brignardello-Petersen, R., Zeng, L., Murad, M.H., Izcovich, A., Morgano, G.P., Baldeh, T., Santesso, N., Cuello, C.G., Mbuagbaw, L., Guyatt, G., Wiercioch, W., Piggott, T., De Beer, H., Vinceti, M., Mathioudakis, A.G., Mayer, M.G., Mustafa, R., Filippini, T., Iorio, A., Nieuwlaat, R., Marcucci, M., Coello, P.A., Bonovas, S., Piovani, D., Tomlinson, G., Akl, E.A., 2022. GRADE guidance 35: update on rating imprecision for assessing contextualized certainty of evidence and making decisions. J Clin Epidemiol 150, 225-242.
